# A Comprehensive Study via Proton Nuclear Magnetic Resonance of a Variety of Omega-3 Lipid-Rich Supplements Available in the Spanish Market: Acyl Group Profile, Minor Components, and Oxidative Status

**DOI:** 10.3390/foods14244217

**Published:** 2025-12-08

**Authors:** Dafne Denise Weinbinder, María J. Manzanos, Patricia Sopelana

**Affiliations:** Food Technology, Faculty of Pharmacy, Lascaray Research Center, University of the Basque Country (UPV/EHU), 01006 Vitoria-Gasteiz, Spain; dafnedenise.weinbinderg@ehu.eus (D.D.W.); mariajose.manzanos@ehu.eus (M.J.M.)

**Keywords:** dietary supplements, omega-3 lipids, ^1^H NMR, acyl groups, lipid classes, tocopherols, sterols, flavour components, oxidation compounds

## Abstract

Dietary supplements are a means of increasing the consumption of beneficial ω-3 lipids. Their composition in acyl groups, the structures supporting them, and their minor component profile can influence their oxidative stability and even their health effects. However, information about all these features is not always provided to consumers. The purpose of this study was to characterise via Proton Nuclear Magnetic Resonance (^1^H NMR) a variety of ω-3 lipid-rich supplements available in the Spanish market, addressing the previously mentioned aspects, together with their oxidative status. Despite not being indicated on the labelling, ethyl esters were found in most fish oil concentrate-based supplements, sometimes as the main lipid class. Furthermore, considerable levels of partial glycerides were present in some samples. Minor components include tocopherols, sterols, terpenic compounds from added flavourings, and vitamin A, many of them with antioxidant and/or bioactive potential. In addition, other unexpected compounds such as BHT and ethanol were detected. Some discrepancies between real sample composition and label information have been noticed regarding the tocopherol profile. Most supplements contained small quantities of hydroperoxides and considerably fewer aldehydes, with significant differences observed among samples, which were not directly associated with their unsaturation degree. These outcomes reinforce the usefulness of ^1^H NMR to provide data about key factors determining the quality of ω-3 lipid-rich supplements in a very simple and fast way.

## 1. Introduction

Omega-3 (ω-3) polyunsaturated fatty acids, especially eicosapentaenoic (EPA, C20:5) and docosahexaenoic (DHA, C22:6), are essential in human nutrition. They are considered to play an important role in human health due to the numerous benefits attributed to them in the prevention of cardiovascular diseases, inflammation, hypertension, or cancer [1,2]. Taking this into account, as well as the often low intake of ω-3 lipids through diet, it is recommended to increase such consumption. To this aim, dietary supplements rich in this type of lipid emerge as a viable option for achieving this goal. The main natural sources of EPA and DHA are marine organisms like fish, krill, or algae [3,4]. In addition, there are terrestrial plant sources of ω-3 lipids like linseed or chia seed oils, rich in α-linolenic acyl groups (ALA, 9,12,15-octadecatrienoic acid, C18:3). In addition, certain ALA-rich oils, like those obtained from echium (*Echium plantagineum*) and ahiflower (*Buglossoides arvensis*), show a considerable content (11–21%) of another less common type of ω-3 acyl group: stearidonic (SDA, 6,9,12,15-octadecatetraenoic acid, C18:4) [5]. This can also be found in seafood lipids [6], but usually at much lower levels (0.5–2% of the total fatty acids). Notably, ALA and SDA act as metabolic precursors from which longer-chain ω-3 EPA and, to a lesser extent, DHA groups can be synthesised [7]. However, the degree of conversion of SDA into EPA is higher than that of ALA [3,8].

Dietary supplements rich in ω-3 lipids differ not only in the nature and proportions of ω-3 groups but also in the structure in which they are supported. In plant- and marine oil-based products, ω-3 groups are mainly in the form of triglycerides (TG), and in the case of krill oils, also in phospholipids [9,10]. Nevertheless, the presence in the market of supplements highly concentrated in ω-3 lipids, obtained from modified fish oils, is becoming increasingly common. Focusing on these latter, it must be noted that, whether specified or not, some of them contain EPA and/or DHA groups in the form of ethyl esters (EE), which are formed during concentration processes [2]. EE can be re-esterified back into TG, giving rise to TG with high proportions of EPA and/or DHA chains. Additionally, diglycerides can also be generated as side products in these re-esterification reactions [2,11,12]. Therefore, different types of acyl group-supporting structures can be found in certain dietary supplements with a high concentration of marine-origin ω-3 lipids. This does not happen in the case of terrestrial plant-derived ω-3 groups, because their natural sources (vegetable oils like linseed, chia, or echium, among others) render higher levels of these types of lipids than fish, so they are not subjected to concentration processes. Microalgae oils can also provide an elevated proportion of highly unsaturated long-chain ω-3 groups, especially DHA [13].

The lipid classes present in commercial supplements can have an impact on two issues directly related to the effect of ω-3 lipids on humans: their bioavailability and their oxidative stability. In this regard, it has been reported that the bioavailability of ω-3 lipids in TG is higher than in EE [9,14,15] and that acyl groups in the EE form seem to be more susceptible to oxidation than in TG [16,17,18]. It is well-known that ω-3 lipids, especially those highly polyunsaturated long-chain ones, are very susceptible to oxidation, which can lead to ω-3 lipid losses and even to the generation of toxic compounds [19]. This susceptibility is influenced not only by their unsaturation degree and, as just mentioned, by their molecular form [9,20], but also by the minor component profile of the supplements containing these lipids. Precisely in order to limit lipid oxidation, compounds with antioxidant ability are commonly added to ω-3 lipid-rich dietary supplements, especially tocopherols, but also others like rosemary extract or ascorbyl palmitate [2,21,22].

Considering the importance of the composition and quality of ω-3 lipid-rich supplements for their health effect, numerous studies have been conducted in recent decades to analyse the products available in the market in several countries all over the world [2,15,21,22,23,24,25,26,27,28,29,30,31,32,33,34,35,36,37,38,39,40,41,42,43,44,45,46,47,48,49,50,51,52]. Most of these investigations have been carried out with supplements containing or obtained from fish oils, although some of them also include samples constituted by krill, algae, or vegetable oils, mainly linseed [2,21,22,25,28,33,35,42,48]. These studies have focused on supplement fatty acid composition and its compliance with the information declared on the label, the fatty acid profile being usually determined by Gas Chromatography (GC) coupled to either a flame ionisation detector (FID) [11,15,23,24,27,29,31,40,41,45,46,47,48,50,51] or a mass spectrometry (MS) detector [2,36,43]. Spectroscopic techniques have also been used for fatty acid analysis, such as mid-infrared Spectroscopy [42,53], Raman Spectroscopy [38], or Proton Nuclear Magnetic Resonance (^1^H NMR) [28,33,42,54,55]. Moreover, the lipid classes present in ω-3 lipid-rich supplements have been the subject of study in several works [2,11,15,38,42,48,51,53], in some of them via ^1^H NMR [12,28,56].

In addition to the study of supplement composition in main components, many studies also tackle the assessment of their oxidative status, another aspect considered crucial for their quality. The oxidation level is generally determined by measuring the peroxide and the *p*-anisidine values, from which the total oxidation value (TOTOX) can be calculated [23,24,25,29,34,45,46,47,48,57]. However, these classical methodologies provide limited information about the specific nature of the oxidation products present in the supplements, which is particularly important in the case of secondary oxidation products like aldehydes, due to their toxic potential [58]. Moreover, according to some authors [21,34], the flavourings added to certain supplements seem to interfere with the *p*-anisidine value measurement, leading to more elevated values. This poses a problem, especially in fish oil-derived non-encapsulated supplements, which usually contain flavouring agents in order to mask their fishy taste. Other means to assess the oxidation level of ω-3 lipid-rich supplements, with different specificities, have also been employed, like Solid-Phase Microextraction followed by GC−MS, which enables the determination of individual volatile oxidation compounds [28], commercial kits to measure alkenal levels [59], the conjugated diene analysis [15], the performance of Rancimat assays at 80 °C to measure induction periods [30], Fourier Transform Raman Spectroscopy [38], or ^1^H NMR for the determination of aldehydes [35]. Furthermore, some researchers have analysed in detail the oxylipin pattern in ω-3 lipid-rich supplements of marine origin by Liquid Chromatography coupled to MS [60,61].

It is worth highlighting that the composition of ω-3 lipid-rich supplements in minor components with antioxidant ability, either naturally present in them or added to limit oxidation, can affect their oxidative stability. Nonetheless, there are very few studies dealing with the determination of supplement minor compounds, and these are centred on cholesterol [11,27], vitamin A [27], and tocols, including vitamin E [2,27,48]. In addition, the occurrence of potentially antioxidant methylated furan fatty acids [62] in marine oil-based supplements has also been studied by some researchers, including the authors of the present study [63,64,65,66].

Taking into account the above background, the purpose of this study is to characterise via ^1^H NMR several ω-3 lipid-rich supplements commercially available in the Spanish market, including samples from different lipid sources and with a wide range of ω-3 group concentrations. The interest is focused on the composition of supplements in acyl groups, lipid classes supporting them, minor components, and oxidation level. The ^1^H NMR technique, a powerful tool for lipid characterisation and oxidation assessment [67,68,69,70,71,72], allows one to obtain all this information simultaneously in a single run, without the need for any sample preparation or modification. To the best of our knowledge, a holistic study addressing all these aspects of ω-3 lipid-rich supplements via ^1^H NMR has not been accomplished before, either with regard to Spanish supplements or from other countries.

The variety of the supplements subject to study, combined with the comprehensive perspective offered by ^1^H NMR, is expected to provide a broad overview of the composition of ω-3 lipid-rich supplements. This will allow for further insight into this type of food product, where knowledge remains especially limited regarding acyl-group supporting structures and minor components with antioxidant and/or bioactive potential. These supplement characteristics can influence the ω-3 lipid oxidative stability and bioaccessibility, so a deeper knowledge of them could contribute to a better understanding of their health effects.

## 2. Materials and Methods

### 2.1. Samples of Study

17 ω-3 lipid-rich dietary supplements of varied nature (S1–S17), 12 encapsulated and 5 non-encapsulated, were acquired in Spanish local markets and through online commerce. They were selected to cover a broad range of ω-3 lipid concentrations and sources: fish lipids (3 supplements comprising natural (non-modified) fish oils and 7 fish oil concentrates, S1–S3 and S11–S17, respectively), vegetable oils (3 samples, S4–S6), microalgae oils (2 samples, S7 and S8), and mixtures of marine and vegetable oils (2 samples, S9 and S10). The main data about their composition, extracted from the information given on their respective labels, are shown in Table 1. Although most samples were sourced from a single batch, two batches (A and B) of some supplements (S9, S12, and S17), acquired at different times, were also analysed. This makes a total of 20 samples studied.

### 2.2. Analysis by ^1^H NMR Spectroscopy

#### 2.2.1. Operating Conditions

The equipment used was a Bruker Avance 400 spectrometer (Bruker Scientific Instruments, Billerica, MA, USA) operating at 400 MHz. Approximately 0.16 g of the liquid matrix from each supplement was dissolved in 400 μL of deuterated chloroform (CDCl_3_) containing tetramethylsilane as an internal reference (Eurisotop, Paris, France), and two spectra were acquired for each supplement. The operating conditions, the acquisition parameters, and the software employed were consistent with those used previously [73]: spectral width 5000 Hz, relaxation delay 3 s, number of scans 64, acquisition time 3.744 s, and pulse width 90°, with a total acquisition time of 8 min 14 s. The selected relaxation delay and acquisition times ensure complete proton relaxation, allowing signal areas to be proportional to the number of protons generating them, thereby enabling their use for quantitative analysis. The experiments were conducted at 25 °C, and the ^1^H NMR spectra were processed using the MestreNova software v16 (Mestrelab Research, Santiago de Compostela, Spain).

#### 2.2.2. Identification and Quantification of Some Types of Compounds and Structures Present in the Liquid Matrix of the Samples

The chemical shifts, multiplicities, and assignments of the ^1^H NMR signals in CDCl_3_ of the main protons of different types of acyl groups/fatty acids susceptible to being present in the samples subject to study and of the various structures that could support acyl groups (TG, partial glycerides, and EE) are displayed in Table 2 [42,68,69,71,74,75,76]. Tables S1 and S2 (Supplementary Materials), in turn, show the chemical shifts, multiplicities, and assignments of some ^1^H NMR signals of certain types of minor components [77,78,79,80,81,82,83,84,85,86,87,88] and oxidation compounds [89,90,91,92,93,94,95], respectively. The areas of some of the signals included in these tables were used to estimate the concentration of the various components in the samples.

The equations employed for the determination of the molar percentages of the main kinds of acyl groups/fatty acids were based on previously developed approaches for vegetable oils [76,96] and fish lipids [71,81], and they are given in the Supplementary Material (Equations (S1)–(S7)). Regarding minor components and oxidation compounds, their concentrations were estimated in millimoles per mole of total acyl groups plus fatty acids (mmol/mol AG + FA) by means of Equation (S8), as in previous studies [97,98]. The standard compounds used for identification and/or quantification purposes are detailed in the Supplementary Material.

### 2.3. Statistical Analysis

The significance of the differences among the data obtained for the various compounds analysed in the supplement samples was assessed by means of one-way analysis of variance (ANOVA) followed by Tukey’s b test at *p* < 0.05. The statistical analysis was performed using SPSS version 28.0.1.0 for Windows (IBM Corp., Armonk, NY, USA).

## 3. Results and Discussion

### 3.1. Information Obtained via ^1^H NMR About Supplement Composition in Acyl Groups and the Structures Supporting Them

Given the complexity of the composition of commercial ω-3 lipid-rich supplements regarding the variety of acyl groups and lipid structures, the ^1^H NMR spectra of standard compounds of different ω-3 lipids, as well as of certain ω-6 lipids that could also be present in these supplements, were analysed prior to studying the samples. The aim was to determine the proportions of acyl groups in the supplement samples as accurately as possible, which required identifying their characteristic spectral signals and assessing potential interferences among them.

#### 3.1.1. Analysis of the ^1^H NMR Spectra of Some ω-3 and ω-6 Lipid Standard Compounds

**Figure 1:**
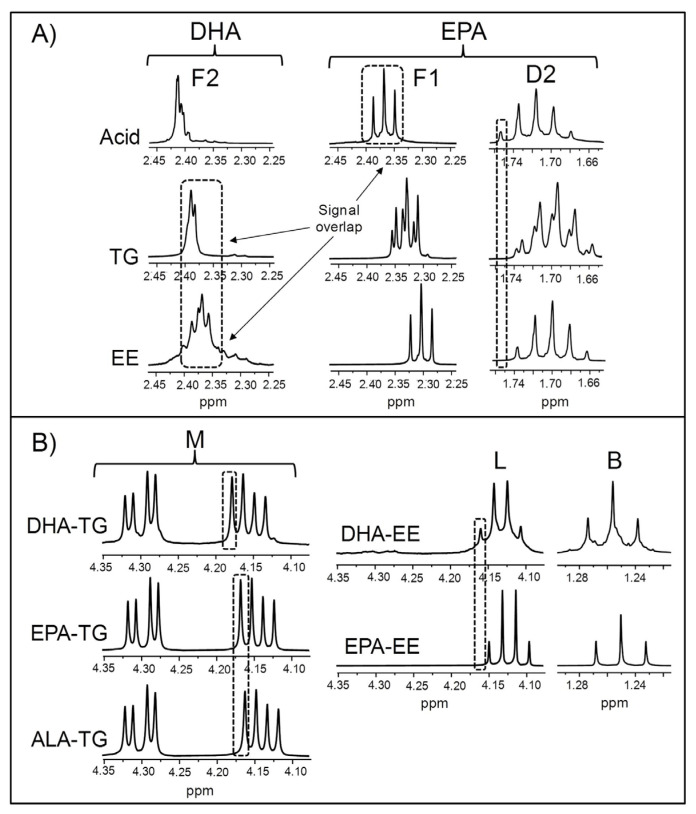
Some enlargements of the ^1^H NMR spectra of certain ω-3 lipid standards, corresponding to: (**A**) the regions where signals F2, F1, and D2 appear in the spectra of DHA (docosahexaenoic) and EPA (eicosapentaenoic) in the forms of acid, triglyceride (TG), and ethyl ester (EE); and (**B**) the regions where some characteristic signals of TG (signal M) or EE (signals L and B) appear in the spectra of DHA and EPA in the forms of TG and EE, as well as in the spectrum of the ALA (α-linolenic)-TG. Signal letters agree with those in Table 2.

As previously mentioned, numerous types of acyl groups can be present in ω-3 lipid-rich supplements. These can be supported in different structures, particularly in modified fish oil-derived supplements that are highly concentrated in EPA and/or DHA groups, so attention must be paid to the shape and chemical shift of their ^1^H NMR spectral signals. This is because they can vary depending on the molecular form of the EPA and DHA chains [75], and this might affect the approaches used for their quantification and/or the accuracy of the results obtained. Bearing this in mind, the ^1^H NMR spectra of EPA and DHA in the forms of acid, TG, and EE were acquired. These are depicted in Figure S1 (Supplementary Materials), while the enlargements of certain spectral regions are displayed in Figure 1.

Figure 1A shows the ^1^H NMR signals of the protons attached to the carbons in α- and β-positions in relation to the carbonyl/carboxyl group in DHA (signal F2, Table 2) and in EPA chains (signals D2 and F1, Table 2), in acid, TG, and EE forms. It must be noted that, as indicated in Table 2, signals F2 and D2 are those used for the quantification of DHA and EPA groups, respectively. The acid form of DHA gives the F2 signal most shifted to lower fields, and the opposite is true for the EE form. Regarding EPA, the same trend is observed in the chemical shift of the F1 signal. As for the D2 signal, a small displacement to lower fields occurs when EPA is in acid form compared with the TG and EE forms. Based on the chemical shifts of the F1 and F2 signals, some inaccuracies due to signal overlap could arise in the quantification of DHA acyl groups if there were simultaneously free EPA groups, as previously noted [75]. These errors could be greater in case DHA groups were in the EE form.

Information about the structures supporting acyl groups can be quickly obtained from the spectral region between 4.08 and 4.34 ppm, where signals due to some protons of the glycerol backbone of TG (signal M) and of the ethyl chain of EE (signal L) appear (Table 2). Figure 1B shows an enlargement of this region in the spectra of DHA-, EPA-, and ALA-TG and of DHA- and EPA-EE, revealing clear differences between the signals of both types of structures. It is also observed that the right double doublet of signal M in DHA-TG is shifted to higher ppm values compared with the other TG. A similar spectral feature is observed in the case of the L signal of DHA-EE compared with that of EPA-EE. Lastly, acyl groups in EE form give a characteristic triplet at approximately 1.25 ppm (signal B, Table 2), due to the methylic protons of the ethoxy group [99].

Acyl groups can also be supported in partial glycerides (mono- and di-glycerides), whose signals have already been described in detail previously [75,76]. Although the proportions of partial glycerides in natural edible oils are usually very small, these can reach high levels in some ω-3 lipid-rich supplements comprising fish oil concentrates [28]. Considering this, it must be remembered that, as Table 2 shows, the F1 signal of 2-monoglycerides (2-MG) overlaps with the characteristic F2 signal of DHA-TG [75] and also with that of DHA-EE. The same can be said of the F1 signal of both 1-MG and 1,3-diglycerides (1,3-DG) and the F2 signal of DHA-EE (Table 2). Consequently, these signal overlaps should be taken into account in samples containing MG and 1,3-DG, especially if their levels are elevated.

**Figure 2:**
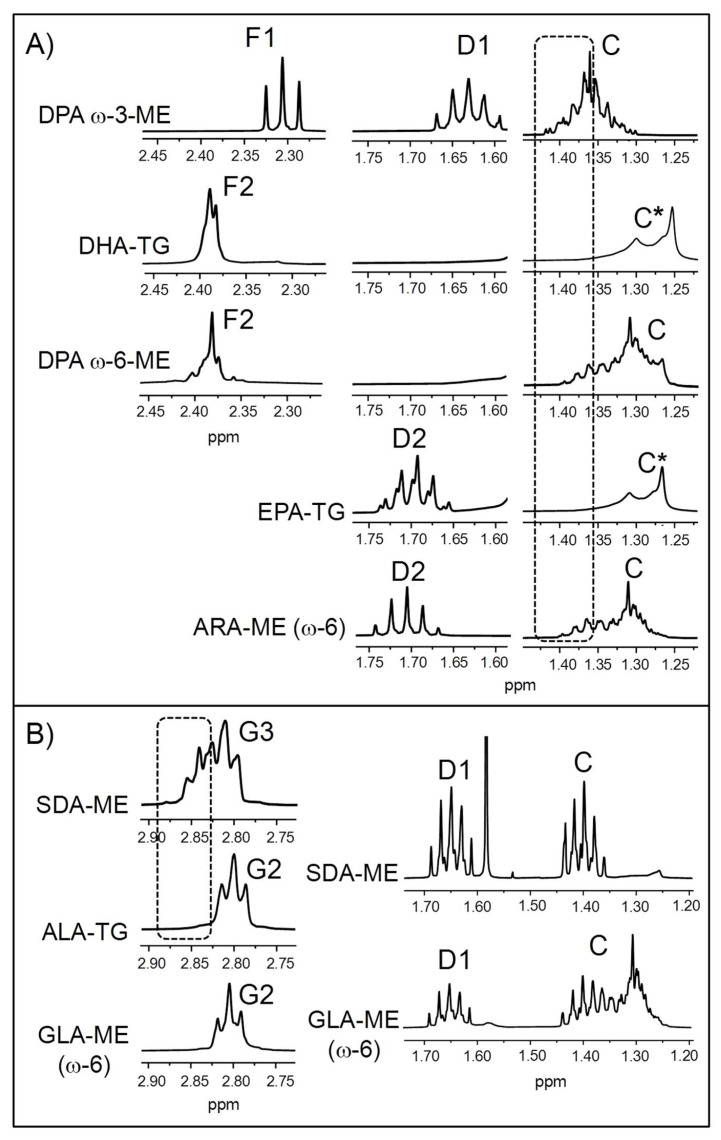
Some enlargements of the ^1^H NMR spectra of standard compounds of (**A**) DPA (docosapentaenoic) ω-3, DPA ω-6, and ARA (arachidonic) (ω-6) methyl esters (ME), together with DHA (docosahexaenoic) and EPA (eicosapentaenoic) in TG (triglyceride) form; and (**B**) SDA- (stearidonic) and GLA (γ-linolenic) (ω-6)-ME and ALA (α-linolenic) in TG form. * The C signal is due to the presence of other types of acyl groups different from DHA/EPA (impurities) in the standard compounds of DHA- and EPA-TG. Signal letters agree with those in Table 2.

Although EPA and DHA groups are the main ω-3 ones present in marine lipids, others like 7,10,13,16,19-docosapentaenoic (DPA, C22:5) can also be found. Some enlargements of the ^1^H NMR spectrum of the DPA ω-3 methyl ester (DPA ω-3-ME) are depicted in Figure 2A (see the full ^1^H NMR spectrum in Figure S2).

As can be seen in Figure 2A, the signal due to the protons in the α-position relative to the carbonyl/carboxyl group of DPA ω-3-ME (F1) does not coincide with that of DHA groups (F2, DHA-TG). Consequently, the potential presence of DPA ω-3 chains in an ω-3 lipid-rich supplement would not affect the quantification of DHA. In contrast, there is a DPA isomer of the ω-6 series (4,7,10,13,16-docosapentaenoic acid; see the full ^1^H NMR spectrum in Figure S2), found in considerable concentrations in some microalgae oils [13,100], whose F2 signal overlaps with that of DHA (Figure 2A). Therefore, this must be borne in mind when quantifying DHA in samples that may contain DPA ω-6 groups. Figure 2A also shows that the spectra of both DPA ω-3- and DPA ω-6-ME exhibit some characteristic methylenic proton signals in the region between 1.25 and 1.43 ppm (signal C), absent in the spectra of DHA and EPA groups [69], which could help to detect their occurrence in ω-3 lipid-rich supplement samples. In addition, DPA ω-3 groups give a multiplet between 1.59 and 1.67 ppm (signal D1) that would slightly overlay with the D2 signal of EPA groups (EPA-TG). However, it must be noted that there can be small differences between the chemical shifts of some signals in ME and TG. In any case, given the slight overlap and the fact that DPA ω-3 groups are generally much less abundant than EPA ones [11,31,48], their presence would not substantially affect EPA quantification.

Another type of acyl group that can interfere with EPA group quantification to a much greater extent than DPA ω-3 is arachidonic (ARA, 5,8,11,14-eicosatetraenoic acid, C20:4, see the full ^1^H NMR spectrum in Figure S2), an ω-6 group present in variable concentrations in fish lipids [101,102]. As previously reported [71], the D2 signal of ARA groups overlaps with that of EPA (Figure 2A), and the slight shiftof the former to lower fields can be due to the fact that it is in ME form and not in TG. However, ARA groups could be identified by part of their C signal, although as Figure 2A reveals, both DPA ω-3 and ω-6 groups give signals in the same spectral region.

An additional sort of ω-3 acyl group that could be present in ω-3 lipid-rich supplements is the above-mentioned SDA (see the full ^1^H NMR spectrum in Figure S2). It is not so widely distributed in nature as other types of ω-3 groups like DHA, EPA, or ALA, but, as indicated in the Introduction Section 1, it appears in significant proportions in echium and ahiflower oils [103,104,105], also used in the manufacture of these kinds of supplements. Some enlargements of the ^1^H NMR spectrum of SDA-ME are presented in Figure 2B. Although its *bis*-allylic proton signal (G3) overlaps to a great extent with that of ALA groups (G2), which are the main ω-3 ones present in vegetable oils, a part of it can be distinguished. This means that the detection and quantification of SDA groups can be accomplished by ^1^H NMR, provided that SDA-rich oils are not mixed with marine oils containing ω-3 groups like DHA and/or EPA, in which case their respective *bis*-allylic proton signals would completely overlap (Table 2).

In addition, SDA-rich oils contain certain proportions of γ-linolenic acid (GLA, 6,9,12-octadecatrienoic acid, C18:3, see the full ^1^H NMR spectrum in Figure S2), a linolenic isomer of the ω-6 series [103,104,105]. Figure 2B reveals that the *bis*-allylic proton signal of GLA-ME (G2) totally overlaps with that of the ALA groups. Hence, in the presence of GLA, the molar percentage of ALA chains cannot be estimated from the G2 signal by means of Equation (S4), unless the proportion of the former is negligible. Finally, SDA and GLA groups also give overlapping signals between 1.35 and 1.45 ppm (signal C) and between 1.6 and 1.7 ppm (signal D1). Notably, these latter would partially overlap with the D2 signal of EPA groups (Figure 2A), so this should be kept in mind when quantifying EPA groups in supplements constituted by mixtures of marine oils with SDA- and/or GLA-containing vegetable oils.

#### 3.1.2. Visual Analysis of the ^1^H NMR Spectra of the Studied Supplements

**Figure 3:**
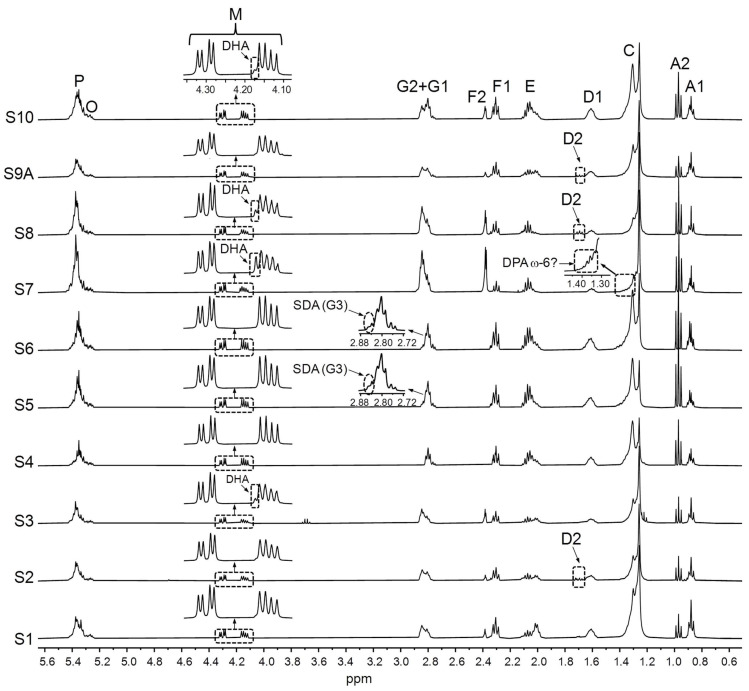
Full ^1^H NMR spectra of the S1–S10 samples, together with the enlargements of certain spectral regions. Signal letters agree with those in Table 2. DHA: docosahexaenoic; DPA: docosapentaenoic; SDA: stearidonic.

**Figure 4:**
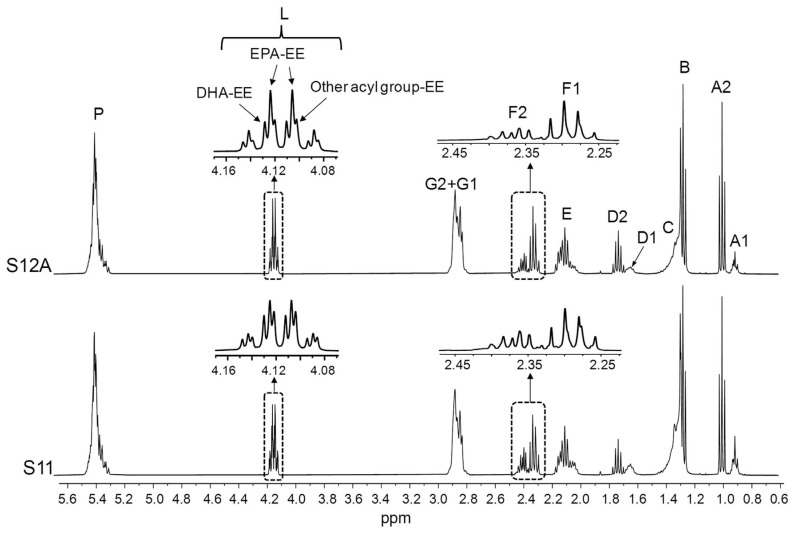
Full ^1^H NMR spectra of the S11 and S12A samples, together with the enlargements of certain spectral regions. Signal letters agree with those in Table 2. DHA: docosahexaenoic; EPA: eicosapentaenoic; EE: ethyl esters.

**Figure 5:**
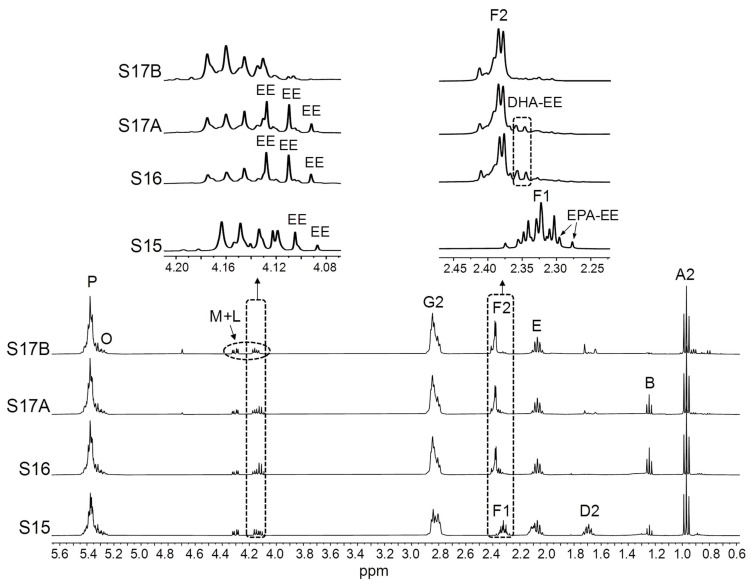
Full ^1^H NMR spectra of the S15, S16, and S17 (A and B) samples, together with the enlargements of certain spectral regions. Signal letters agree with those in Table 2. DHA: docosahexaenoic; EPA: eicosapentaenoic; EE: ethyl esters.

**Figure 6:**
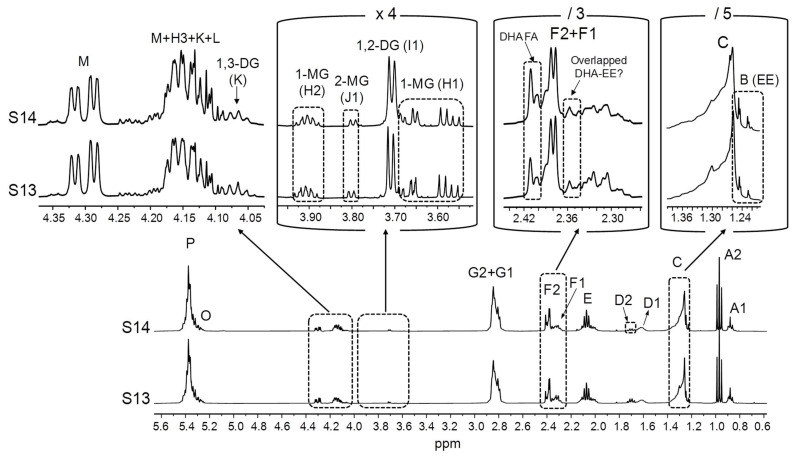
Full ^1^H NMR spectra of the S13 and S14 samples, together with the enlargements of certain spectral regions. The degree of increase or reduction in some of the spectral excerpts in relation to the one on the left is indicated to be able to compare the intensities of the various signals: [×4]: 4-time enlargement; [/3] and [/5]: 3- and 5-time reductions, respectively. Signal letters agree with those in Table 2. DHA: docosahexaenoic; EE: ethyl esters; DG: diglycerides; MG: monoglycerides; FA: fatty acids.

Considering all the above, a great deal of useful information can be obtained from the simple visual observation of the ^1^H NMR spectra of the supplement liquid matrix. This can be verified in Figure 3, Figure 4, Figure 5 and Figure 6, which show the full ^1^H NMR spectra of the studied samples, together with the enlargements of certain spectral regions. By examining the spectral excerpts between 4.10 and 4.35 ppm in the S1–S10 samples (Figure 3), it is possible to know, at a glance, that they are constituted by TG (signal M). In addition, in the S3, S7, S8, and S10 samples, the presence of variable proportions of DHA groups can be inferred from the left shoulder of the part of the M signal between 4.10 and 4.19 ppm.

Figure 4 evidences that the S11 and S12 samples, with a high proportion of ω-3 lipids (Table 1), are constituted by EE (signal L). Given that DHA-EE signals are slightly shifted to the left in relation to those of EPA-EE (Figure 1B), it is deduced that EPA groups are more abundant than DHA ones and that the proportion of DHA in comparison with that of EPA is higher in S11 than in S12. The same can be inferred from the shape, chemical shifts, and intensities of the F1 and F2 signals. Furthermore, the L signal multiplicity points to the presence of EE of other types of acyl groups different from DHA and EPA.

The analysis of the spectral region between 4.08 and 4.19 ppm in the S15, S16 and S17 samples (Figure 5) allows one to know that, as well as TG, they contain variable proportions of EE. This is also clearly observed for DHA and EPA groups from F2 and F1 signals, respectively. Considering that, according to label information, these samples have a high ω-3 group concentration (Table 1), the presence of EE might be explained by an incomplete re-esterification of acyl groups from EE to TG during the concentration of ω-3 lipids [2,11]. A simultaneous presence of TG and EE in ω-3 lipid-rich supplements available in the French market was also reported by Pasini and coworkers [48]. Thus, even supplements stated to contain TG, such as S15, S16, and S17 (Table 1), have been found to contain a certain proportion of EE, consistent with reported data [106]. Figure 5 also shows that the level of EE in the two batches of the S17 sample was quite different. However, despite the occurrence of EE in ω-3 lipid-rich supplements could affect their oxidative stability and bioavailability, information about the presence of these lipid structures is not given on the label of any of the samples studied, even when EE is the main acyl group-supporting structure, as in the S11 and S12 samples. This lack of information about lipid classes has also been noticed in ω-3 lipid-rich supplements from the Brazilian [42] and German markets [2].

With regard to the S13 and S14 samples, their high level of partial glycerides stands out, although according to the label, in the former, DHA is in its natural TG form (Table 1). This can be observed in Figure 6, where spectral signals of all the types of partial glycerides are clearly observable (H1 and H2 of 1-MG, I1 of 1,2-DG, J1 of 2-MG, and K of 1,3-DG). The intensities of these signals reveal that DG are in higher concentrations than MG. Similarly to what has been argued in relation to EE, the presence of MG and especially of DG in these samples could be the result of an incomplete TG re-esterification during the ω-3 lipid concentration process [2,11], as both of them are very rich in ω-3 groups (Table 1). Indeed, levels of DG up to 15% were found by Damerau and coworkers [28] in Finnish supplements presumably made up of re-esterified TG. Figure 6 also shows that partial glycerides are accompanied by a noticeable proportion of DHA fatty acids (FA). In this regard, it should be noted that an elevated proportion of partial glycerides and particularly FA in ω-3 lipid-rich supplements could negatively affect their oxidative stability, as they are considered to be more susceptible to oxidation than TG with equivalent unsaturation degrees [20,107]. Despite this, the labels of the S13 and S14 samples do not provide any information about the presence of partial glycerides or FA. In addition, these supplements also contain EE. Although their L signal (Table 2) is difficult to distinguish in the ^1^H NMR spectra of these samples, the presence of EE can be inferred from the B signal (Table 2), partially overlapped with signal C (Figure 6). Taking into account that, according to the label information (Table 1), DHA groups are the most abundant in the S13 and S14 samples, it would be expected that DHA-EE would be in higher concentrations than EE of other acyl groups. In that case, an overlay would occur among the F2 signal of DHA-EE and the F1 signals of DG, and/or MG of other types of acyl groups and potentially present fatty acids different from DHA, which could affect DHA quantification.

As for the supplements comprising ahiflower and echium oils (S5 and S6, respectively, Table 1), the presence of SDA groups can be deduced from the ^1^H NMR spectral excerpts in Figure 3. Lastly, the detailed analysis of the spectral region between 1.33 and 1.41 ppm approximately in the microalgae oil-based S7 sample (Figure 3), suggests the presence of DPA ω-6 groups.

**Figure 7:**
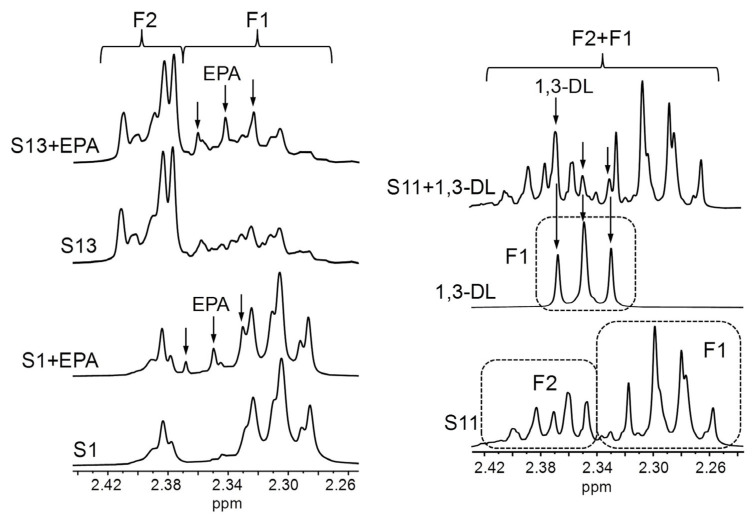
Some enlargements of the regions where signals F1 and/or F2 appear in the ^1^H NMR spectra of the S1 and S13 supplements, these samples enriched with free EPA (eicosapentaenoic) (S1 + EPA and S13 + EPA), the S11 supplement, the standard compound of 1,3-dilinolein (DL), and the S11 sample enriched with 1,3-DL (S11 + 1,3-DL). Signal letters agree with those in Table 2.

In the context of this analysis, it must be noted that the chemical shifts of the signals given by pure compounds dissolved in CDCl_3_ can differ from those observed in real lipid samples [54,108]. Therefore, in view of the variety of acyl group-supporting structures present in the supplements most concentrated in ω-3 lipids (S11–S17, Table 1), particularly in the S13 and S14 samples (Figure 6), it was investigated whether certain potential signal overlaps deduced from the spectra of individual pure compounds, either in this work (Figure 1A) or in others conducted previously [75], would also occur in real samples. For this purpose, pure EPA in acid form was added to the S13 sample and to another one (S1), basically containing only TG, to analyse the extent of the overlap between free EPA and DHA acyl group signals. In addition, the S11 sample, constituted by EE (Figure 4), was supplemented with 1,3-dilinolein (1,3-DL, full ^1^H NMR spectrum in Figure S3) to assess the interference between 1,3-DG and DHA-EE signals. As shown in Figure 7, when free EPA is added to the supplement samples, its F1 signal shifts slightly towards higher fields compared with the pure compound (Figure 1A). Consequently, it does not overlay with the F2 signal of DHA groups bound to TG, whereas such overlap would occur in the case of DHA-EE. In contrast, when 1,3-DL is added to the S11 sample, the chemical shift of its F1 signal remains unchanged in comparison with the pure compound spectrum. Hence, its overlap with the F2 signal of DHA-EE would also take place in real samples. Taken together, these results suggest that, in supplement samples containing DHA-EE and substantial proportions of 1,3-DG, such as S13 and S14 (Figure 6), DHA group quantification could be subject to a certain degree of inaccuracy.

#### 3.1.3. Proportions of the Various Types of Acyl Groups in the Samples Studied

##### Some Considerations Related to the Determination of the Molar Percentages of Certain Kinds of ω-3 Acyl Groups in Some Supplements

At the sight of all the above, some considerations should be made in relation to the quantification of ω-3 acyl groups in certain supplements. These refer mainly to those samples likely to contain DPA ω-6 groups, such as S7, and to the samples rich in SDA groups (S5 and S6). In the first case, the molar percentage data obtained from the integration of the F2 signal (Table 2) will correspond to the sum of DHA and DPA ω-6 groups. As a result, the DHA proportion will have to be estimated from the total of ω-3 groups by subtracting the contribution of EPA in case it was present, assuming that the proportion of other types of ω-3 groups different from EPA is comparatively much lower [11,31].

With regard to the S5 and S6 samples, the determination of the molar percentages of SDA and ALA groups can be made as follows:

SDA group % = 100 × (A_G3_/3A_F1_), where A_G3_ and A_F1_ are the areas of the G3 and F1 signals, respectively (Table 2), A_G3_ being estimated by using an area conversion factor calculated from the spectra of the SDA-ME standard.

ALA group % = Total ω-3 group % − SDA group %, the Total ω-3 group % being estimated by means of Equation (S1).

Similarly, in supplements comprising mixtures of ALA-rich and marine oils such as S10, the ALA group molar % must be calculated by subtracting the corresponding percentages of specific ω-3 groups (DHA and/or EPA) from the total ω-3 group %.

Lastly, it is worth remembering that when quantifying EPA groups, a certain contribution of ARA cannot be discarded, as their respective D2 signals overlap (Figure 2A). Regarding this issue, variable amounts of ARA groups relative to those of EPA can be found in different fish species [101,109]. Thus, whereas the proportion of ARA compared with that of EPA groups is very small in cod liver oil (S1 sample) [108,110,111], in tuna oil (S3 sample), ARA can reach levels similar to or even higher than those of EPA [112,113]. Notwithstanding, signals in the spectral region between 1.35 and 1.40 ppm attributable to ARA groups were not noticeable in the S3 sample. Actually, the only supplement in which signals tentatively assignable to ARA groups are distinguishable in its ^1^H NMR spectrum is S15 (Figure S4), the richest in EPA groups (Table 1). In addition, as deduced from Figure 2, the EPA amount might be somewhat overestimated in the S9 sample due to the presence of GLA group-containing borage oil (Table 1).

##### Data Obtained

The molar percentages of the main types of acyl groups present in the supplements are given in Table 3. The lowest proportions of ω-3 groups are observed in the samples containing fish oils (S1–S3) and a mixture of fish and borage oils (S9), with values varying between 25.0% in S1 and 33.8% in S3. The S3 sample, manufactured with tuna oil, shows a higher proportion of DHA than of EPA groups, in accordance with the reported data for this oil [112,113]. The opposite is true for the S2 and S9 samples, whereas in S1, the EPA and DHA molar percentages are of the same order, consistent with cod liver oil composition data [81,110,111].

Higher levels of ω-3 lipids were found in the samples comprising vegetable and/or microalgae oils (S4–S8 and S10), with values ranging from 44.0% to 60.6% in the S6 and S5 samples, respectively. Regarding the supplements containing SDA-rich oils (S5 and S6), their SDA proportions match well with reported data [103,104,105]. Microalgae oils (S7 and S8 samples), despite coming from the same species, *Schizochytrium*, present very different EPA and DHA group molar percentages, in such a way that the S7 sample contains almost exclusively DHA, whereas both DHA and EPA are present in S8. The occurrence of DPA ω-6 groups in the S7 sample, which was noted by observing its spectrum (Figure 3), is confirmed by data in Table 3, as the DHA + DPA ω-6 groups molar percentage (66.0%) is noticeably higher than that of total ω-3 lipids (57.0%). The difference (9.0%) will correspond to DPA ω-6 groups, which is in the range found in the *Schizochytrium* species microalgae lipids [100].

Finally, there is a group of samples with very high levels of ω-3 lipids (between 66.7% in S11 and 96.7% in S17A). In agreement with the information extracted from the visual exam of the spectra of the S11 and S12A samples (Figure 4), EPA groups are more abundant than DHA ones, their proportions being more balanced in S11 than in S12. Conversely, S13 and S14 samples show considerably higher concentrations of DHA than of EPA groups. As for the S15, S16, and S17 samples, the richest in ω-3 lipids, they were composed almost entirely of a single type of ω-3 group: EPA in S15 and DHA in S16 and S17, consistent with label information (Table 1).

Regarding variability between batches, significant differences (*p* < 0.05) were found in the DHA group proportion for the S17 sample and in that of EPA for S9 and S12. Furthermore, significant divergencies were observed in the molar percentages of the total ω-3 groups for the most concentrated samples (S12 and S17).

By comparing the total of ω-3 lipids with the sum of the DHA and EPA proportions in the marine oil-based supplements, the presence of other types of ω-3 groups can be inferred. As Table 3 shows, their molar percentage varies between 0.5% in the S15 sample and 14.5% in S14. The high value in the latter could be due to an underestimation of the DHA group proportion, as part of the F2 signal of the DHA-EE potentially present in the S14 sample (Figure 6) was not taken into account for DHA group quantification (see Table 3) due to the high degree of signal overlap in this region. The molar percentages of other ω-3 groups in the samples comprising natural fish oils are of the same order as those found via ^1^H NMR in cod liver oils [81] and fish oil-based dietary supplements [54]. Considering available data on the composition of ω-3 lipid-rich supplements [11,31,48] and fish lipids [7,114], DPA ω-3 groups would be expected to be the main contributors to the pool of other ω-3 groups different from EPA and DHA. Indeed, signals tentatively assignable to DPA ω-3 groups are noticeable in the ^1^H NMR spectra of S2, S11, and S12B samples (Figure S5).

Except for the S7 sample (microalgae oil) and those most concentrated in ω-3 lipids (S14–S17), the remaining samples contain detectable levels of diunsaturated ω-6 acyl groups, mainly linoleic. These are more abundant in the supplements made of vegetable oils (S4–S6) and mixtures of vegetable and marine oils (S9 and S10), with contents ranging from 9.6% in the S10 sample to 14.4% in S4 and S6.

Notably, most of the supplements comprising or manufactured with fish oils present small proportions of ω-1 acyl groups, varying between 0.1% in the S3 and S16 samples and 2.4% in S2. These values are in agreement with ^1^H NMR reported data on fish lipids [71] and fish oil supplements [54]. The ω-1 lipid concentrations are generally lower in the fish oil concentrate-based samples, not being detected in S17. This suggests a loss of ω-1 groups in the ω-3 lipid concentration processes. Although knowledge about the potential bioactivity of this type of lipid, found in phytoplankton and diatoms [115], is scarce, a potent plasma TG-lowering function of 6,9,12,15-hexadecatetraenoic acid (HDTA, C16:4 ω-1) has been reported in mice [116]. Therefore, the presence of ω-1 groups in ω-3 lipid-rich supplements could be valuable.

As can be deduced from the total unsaturated group proportions in the various samples, the supplements composed of natural fish and microalgae oils (S1–S3, S7, and S8), together with the S9 sample, are richer in saturated groups than those containing exclusively vegetable oils and fish oil concentrates. Precisely, the low or negligible content of saturated fatty acids in ω-3 lipid concentrates is perceived as an advantage compared to other types of marine-origin ω-3 lipid-rich supplements [117].

Although a good correlation has generally been reported between acyl group percentages calculated via ^1^H NMR and methods like GC–MS [118] or GC–FID [74,108], slight deviations have been observed in these studies. In addition, as discussed earlier, some acyl groups, such as DHA and DPA ω-6 or EPA and ARA, give overlapping signals, so their amount must be given jointly. Bearing all this in mind, an assessment has been made of the agreement between the acyl group data obtained in the present study and the information extracted from the supplement labels. To this aim, the percentages in weight of certain types of ω-3 groups were taken or calculated from the supplement label when possible. They are given in Table 4, together with the differences between these data and those resulting from the ^1^H NMR analysis (Table 3), expressed as units of percentage. The precise quantities of ω-3 lipids were not specified in several supplements, with only minimum or guaranteed amounts given in the S10, S13, S14, and S16 samples.

It is worth noting that the molar percentages of SDA determined via ^1^H NMR are in quite satisfactory agreement with those declared on the labels of the S5 and S6 samples, coinciding almost exactly in the latter. Therefore, it can be said that the proportion of this acyl group in SDA-rich oils can be determined adequately by ^1^H NMR. Although SDA groups have been quantified in fish oil samples by ^13^C NMR [54], as far as we know, this is the first time that SDA molar percentage data are given using ^1^H NMR.

In relation to what was discussed in previously, the presence of GLA groups in the S9 sample does not appear to have affected EPA quantification, as the data obtained are very similar to those stated on the label. However, in line with what was mentioned in this same section, in the S13 and S14 samples, the DHA group proportions seem to have been underestimated, as none of them reach the minimum established for these supplements. Despite this, it could be said that, overall, the results found for most of the samples included in Table 4 show fairly good agreement with the information provided on their labels. Thus, the observed deviations fall within the range reported by other authors for the differences between data obtained via ^1^H NMR and GC−FID [108,119]. However, greater upward variations are observed for DHA, EPA, and total ω-3 groups in the S3 sample, and particularly for EPA in S8, S12, S15, and S17. In the case of the S15 sample, the presence of ARA groups (Figure S4) could account to some extent for the increased value of EPA molar percentage. With regard to the minimum contents of ω-3 lipids established in some samples, they are surpassed in most cases.

### 3.2. Information About Supplement Minor Components

In addition to the acyl group composition data, the ^1^H NMR analysis provides information about certain minor components present in the supplements. Indeed, compounds of varied nature have been detected, collected in Table 5 and Table 6.

#### 3.2.1. Tocopherols

It is well-known that tocopherols are the main components with attributed antioxidant capacity naturally present in vegetable oils and one of the main classes of antioxidant additives used in food products like the supplements studied (Table 1). As Table 5 shows, tocopherols were detected in almost all supplements (except for S1 and S17). Among these, alpha-tocopherol (α-T), found in less than half of the samples, was generally present in the highest concentrations due to external addition (Table 1), particularly in the S4 sample. In this regard, it must be remembered that a prooxidant behaviour of α-T in oils, especially at elevated concentrations, has been evidenced under accelerated storage conditions [73,120].

Unlike α-T, gamma-tocopherol (γ-T) was present in most samples. Overall, its concentration was higher in the marine oil-based supplements enriched with added tocopherols (Table 1) than in those comprising vegetable oils, with a significant difference (*p* < 0.05) in γ-T content between the two batches of the S12 supplement. Furthermore, in the γ-T-containing samples, delta-tocopherol (δ-T) was also detected, in lesser concentrations than γ-T. Pasini and coworkers [48] also found lower levels of δ-T than of γ-T in most of a series of ω-3 lipid-rich supplements sold in the French market.

Among the supplements comprising vegetable oils, none of them fortified with γ-T-containing tocopherol mixtures (Table 1), the S6 sample (echium oil) exhibits a higher level of natural γ-T than S4 and S5 (linseed and ahiflower oils, respectively). This matches well with the findings of Carlini and coworkers [103], who reported a γ-T concentration almost double in echium than in linseed oil.

It must be noted that, although α-T was the tocopherol declared in the S9 sample (Table 1), γ-T and δ-T were present in one of the batches studied (S9B), whereas α-T was not detected. In this same line, although α-T was the only type of tocopherol stated on the label of the S3, S8, and S14 samples (Table 1), γ- and δ-T were also found, suggesting the addition of a tocopherol blend. Similar discrepancies between label information and supplement composition were observed by Zartmann and coworkers [2]. Lastly, despite the label of the S17 sample declaring it to contain added δ-T (Table 1), it could not be detected due to the presence of unknown overlapping signals in the same ^1^H NMR spectral region.

#### 3.2.2. Sterols

The knowledge of the sterol profile of ω-3 lipid-rich dietary supplements deserves attention due to the properties and health effects of these minor components. While cholesterol, the primary sterol found in animal lipids, may contribute to cardiovascular disease [121], plant sterols have been attributed several health benefits, like a cholesterol plasma level-lowering action and potential anti-cancer effects, among others [122]. As it is known, the ^1^H NMR analysis does not allow one to find out the exact identity of some 4-desmethylsterols due to the overlapping of their respective signals at 0.68 ppm. This is the case for cholesterol, β-sitosterol, ∆5-campesterol, and ∆5-avenasterol (Table S1). However, considering the oil source of the various supplements, one could assume that the signal at 0.68 ppm will correspond mainly to cholesterol in the spectra of fish oil-based samples and to phytosterols of varied nature in those of vegetable oils. As for the supplements constituted by mixtures of marine and vegetable oils (S9 and S10) and those containing microalgae oils (S7 and S8), both cholesterol and phytosterols could be present [123,124]. The concentrations of some sterols detected in the supplements studied are given in Table 5.

The samples manufactured from natural fish oils (S1–S3) generally have higher cholesterol concentrations than most fish oil concentrates (see data on sterols giving a signal at 0.68 ppm), some of them devoid of detectable cholesterol levels. These findings suggest that the processes used for the concentration of ω-3 lipids could help to reduce cholesterol intake in comparison with non-concentrated supplements.

In relation to the supplements containing only vegetable oils, cycloartenol and 24-methylenecycloartanol, characteristic 4,4-dimethylsterols of linseed oil [125,126], were found in free and esterified forms in the S4 sample (see data on DiMe-St). Table 5 also reveals that the sample comprising echium oil (S6) is the richest in 4-desmethylsterols, giving a signal at 0.68 ppm (β-sitosterol and/or ∆5-campesterol and/or ∆5-avenasterol), and also in ∆7-avenasterol.

As far as microalgae oil samples are concerned (S7 and S8), notable differences in their sterol profile can be noticed, in addition to those observed in their acyl group composition (Table 3). Whereas in the S8 sample the amount of stigmasterol stands out, S7 is richer in 4-desmethylsterols giving a signal at 0.68 ppm. Although, as mentioned previously, it is difficult to know which specific sterols are contributing to this signal, it is worth noting that cholesterol has been found to be one of the most abundant sterols in microalgae lipids from the *Schizochytrium* species. The presence of stigmasterol has also been reported in these lipids, but in much lower amounts [123,124].

Regarding the samples constituted by mixtures of vegetable and marine oils, a considerable level of 4-desmethylsterols giving a signal at 0.68 ppm is observed in S9, with a significant difference (*p* < 0.05) between the two batches studied. The sterol profile of the S10 sample seems to be in agreement with the presence of linseed oil (Table 1), which, as previously mentioned, is rich in cycloartenol and methylenecycloartanol (DiMe-St) [125,126].

#### 3.2.3. Other Types of Minor Components of Varied Nature

In addition to tocopherols and sterols, which are present in most of the studied samples, other minor compounds have been detected only in certain supplements (Table 6). As mentioned in Section 2.2.2, their ^1^H NMR signals are given in Table S1. The majority of them originate from the added lemon and mint aromas (Table 1). In agreement with the information given in Table 1, these flavour components are predominantly found in the non-encapsulated supplements (S2, S3, S7, S12, and S17), but also in the S10 sample. Limonene, a cyclic monoterpene, was detected in all the non-encapsulated supplements, most of which also contained geranial and neral; these are the *E*- and *Z*-isomers, respectively, of citral. Limonene concentration varies considerably, ranging from 0.5 mmol/mol AG + FA in the S3 sample to 94.2 mmol/mol AG + FA in S17B. Much lower levels are generally observed for geranial and neral, with the concentration of the former slightly higher than that of the latter, consistent with the composition of lemon peel essential oil [127]. β-Pinene, another monoterpene characteristic of this essential oil, was also found in the S2, S12, and S17 samples. In addition, a monoterpene alcohol derived from mint aroma, menthol, was detected in the S10 and S17 samples, in accordance with mint flavouring addition (Table 1). Whereas the concentrations of most of the detected flavour components are similar in the two batches of the S12 sample, significant differences (*p* < 0.05) of around three times are observed in their levels between the two S17 supplement batches. It should be noted that the majority of these flavour compounds have been attributed antioxidant ability [128,129], so their presence and levels could influence the supplement’s oxidative stability.

Apart from aroma-derived compounds, other types of minor components were detected only in a few samples. One of these is vitamin A (retinyl esters), exclusively found in the supplement constituted by cod liver oil (S1). Furthermore, BHT (butyl-hydroxytoluene) was present in the S7 sample. It is a synthetic antioxidant food additive, but it may also be present in edible oils and fats due to environmental contamination or migration from materials that come into contact with them. Moreover, certain algae are also capable of synthesising BHT naturally [130].

**Figure 8:**
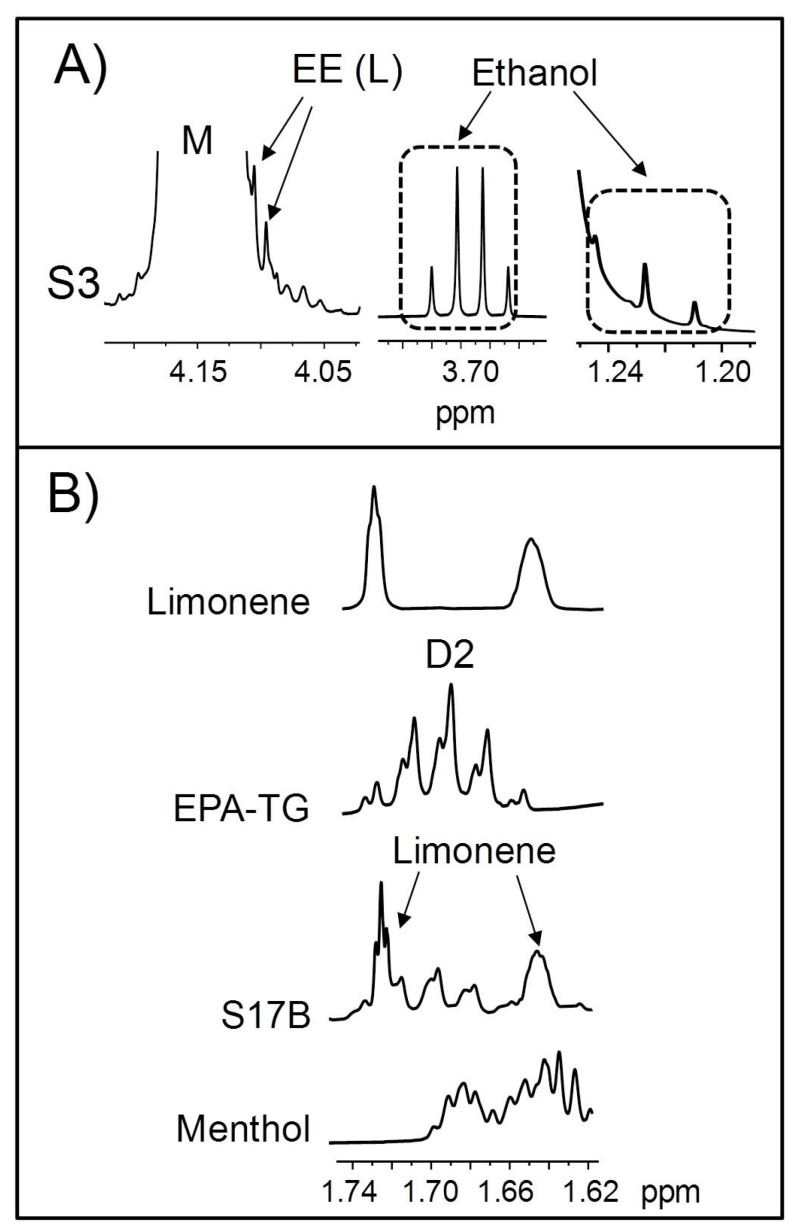
Some enlargements of the ^1^H NMR spectra corresponding to (**A**) the regions where the L signal of ethyl esters (EE) and ethanol signals (Table S1) appear in the ^1^H NMR spectrum of the S3 sample; and (**B**) the region where EPA (eicosapentaenoic) D2 signal appears in the ^1^H NMR spectra of the S17B sample and of the standard compounds of EPA-TG, limonene, and menthol. TG: triglyceride. Signal letters agree with those in Table 2.

Lastly, the presence of ethanol in the S3 sample is worthy of note (Figure 8A), as it is not a common component in natural fish oils. In the context of ω-3 lipid-rich dietary supplement manufacturing, ethanol is used in ethanolysis reactions aimed at converting the acyl groups of fish oils into EE [131]. Precisely, small signals that could be tentatively attributable to these structures can also be noticed in the ^1^H NMR spectrum of this sample (see L signal in Figure 8A), overlapped with the M signal of TG. Therefore, given the simultaneous presence of ethanol and EE, it could be thought that the S3 sample—allegedly manufactured with tuna oil—was accidentally contaminated with a partially ethylated oil intended for a different process. In this regard, Sullivan and coworkers [106] reported the presence of EE in natural fish oils, attributing this to either accidental or deliberate contamination of the product. However, to the best of our knowledge, the presence of ethanol in commercial ω-3 lipid-rich supplements had not been previously described.

As a final remark, it is worth noting that some signals of certain minor components overlap with those used for the determination of some kinds of acyl groups (Table 2 and Table S1). Consequently, their contribution must be subtracted to make an accurate quantification of the acyl group molar percentages. This is particularly important if these compounds are in high concentrations, as is the case with limonene and, to a lesser extent, menthol in the S17B sample (Table 6). The overlay in this sample spectrum of one of the limonene signals with that used for the quantification of EPA (D2) can be clearly observed in Figure 8B. Furthermore, this figure reveals that menthol also gives signals that partially overlap with those of EPA groups.

### 3.3. Information About Supplement Oxidation Level

Table 7 provides data about the presence of certain types of oxidation products in the studied samples. This shows that most of them contain small amounts of primary oxidation compounds with a hydroperoxy group and/or with a hydroxy one associated with conjugated dienic systems with *Z,E*-isomerism (HPO-c-*Z,E*-dEs and HO-c-*Z,E*-dEs, respectively).

The lowest concentrations of HPO-c-*Z,E*-dEs are observed in the samples comprising natural fish oils (S2 and S3), which are the least concentrated in ω-3 groups (Table 3), in the linseed oil-based sample (S4), in one of the microalgae oil supplements (S8), and in the S13 sample, these two latter being quite rich in ω-3 lipids (57.6% and 68.1%, respectively). Conversely, the highest levels correspond to one of the most unsaturated samples, S17B (91.8% of ω-3 groups). The presence of HO-c-*Z,E*-dEs in the S4 sample in more elevated concentrations than HPO-c-*Z,E*-dEs could be related to the high level of α-T in this sample (Table 5), as it can favour the generation of this kind of oxidation derivatives [73,120].

**Figure 9:**
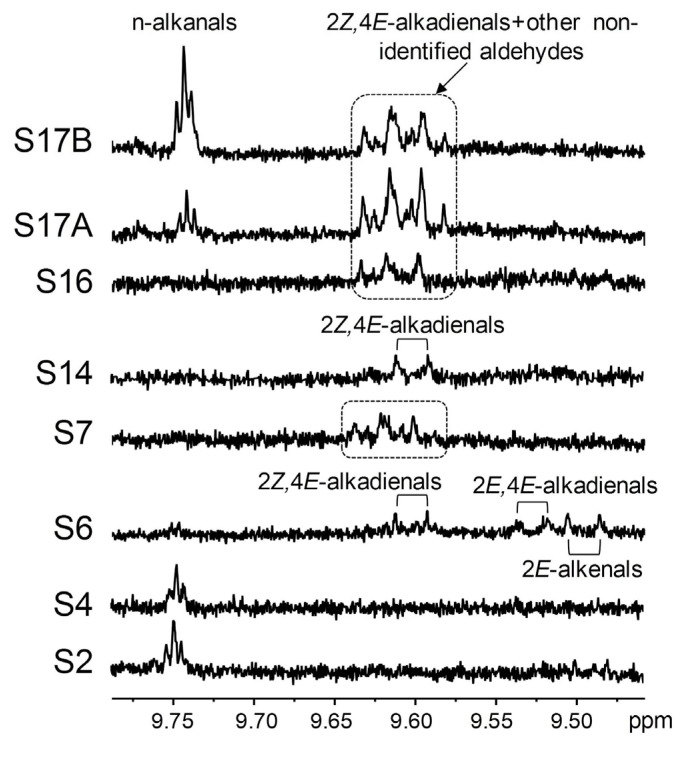
Enlargements of the region of the ^1^H NMR spectra of some supplement samples where aldehydic signals appear. The spectral excerpts have been plotted at a fixed value of absolute intensity to be valid for comparative purposes.

Aldehydes (secondary oxidation derivatives) were detected only in a few samples. These include n-alkanals in S2, S4, and S17; 2*E*-alkenals, 2*E*,4*E*- and 2*Z*,4*E*-alkadienals in S6; and 2*Z*,4*E*-alkadienals in S14. Jansson and Kay [35] also found n-alkanals and 2*E*-alkenals in ω-3 lipid-rich supplements of marine origin via ^1^H NMR. Furthermore, some non-identified aldehydic signals were observed in the S7, S16, and S17 samples, all of them very rich in DHA groups (Table 3). Some of them could correspond to 2*Z*,4*E*-alkadienals (Table S2), but they overlap with others not present in the rest of the samples. Figure 9 shows the aldehydic signals in the various supplements. Notably, potentially toxic oxygenated α,β-unsaturated aldehydes like 4-hydroperoxy-, 4-hydroxy-, and 4,5-epoxy-2*E*-alkenals [94] were not detected in any of the studied samples.

The aldehyde concentrations are noticeably lower than those of HPO/HO-c-*Z,E*-dEs, with the highly unsaturated S17 sample exhibiting the most elevated levels. This may suggest that the samples are in the early stages of oxidation, although it might also be possible that volatile aldehydes had been removed during the supplement manufacturing process [9,61]. However, the unsaturation degree of the samples does not seem to be the only factor affecting their oxidation level, as significant differences (*p* < 0.05) are not observed in the HPO-c-*Z,E*-dE level between samples with very different ω-3 lipid proportions, such as S6 (44.0%) and S16 (92.7%). Moreover, oxidation compounds were not detected in the S12 sample despite its high level of ω-3 groups (75.3% in S12A and 71.8% in S12B).

Interestingly, although the S13 and S14 samples contain high proportions of partial glycerides and fatty acids (Figure 6), making them more prone to oxidation, they do not show a particularly high oxidation level compared with the rest of the samples, even with similar unsaturation degrees.

## 4. Conclusions

The outcomes of this study reinforce the great potential of the ^1^H NMR technique for the study of ω-3 lipid-rich supplements, being able to provide data about the most relevant aspects related to their quality in a very simple and fast way, without the need for any sample pre-treatment. In addition to the ω-3 supplement composition in the various kinds of acyl groups and the structures supporting them, the ^1^H NMR analysis enables the obtaining of valuable information about a wide variety of minor components, such as tocopherols, sterols, and flavour components of terpenic nature, some of them considered of interest due to their antioxidant and/or bioactive potential. Furthermore, ^1^H NMR allows one to assess the oxidative status of these types of food products. All this knowledge is very important because the profile of acyl groups, their presence in TG or EE form, the levels of potential antioxidant compounds, or the nature and abundance of oxidation products are factors influencing the quality of dietary ω-3 lipid-rich supplements and their effect on human health.

The analysis via ^1^H NMR has some limitations for the exact determination of the molar percentages of certain types of acyl groups and the concentrations of some minor components, which will depend on the composition of each specific sample. However, the ^1^H NMR technique provides a great deal of information with a speed and simplicity that is impossible to achieve by other means. Moreover, due to the varied composition of these ω-3 lipid-rich supplements, this untargeted approach of the ^1^H NMR analysis is particularly useful, since it is not focused only on specific components but can comprise all those included in the sample, as long as they give distinguishable ^1^H NMR signals.

The findings of this research have revealed the presence of varying levels of EE in the most concentrated ω-3 lipid-rich supplements, even when some of them only refer to TG on their label. Considering that the structures supporting ω-3 acyl groups can influence their oxidative stability, as well as their bioavailability, this information should be provided by manufacturers, particularly when supplements comprise almost exclusively EE. Furthermore, the ^1^H NMR analysis has allowed the detection of some mismatches between real sample composition and label information in relation to the tocopherol profile, as well as the presence of unexpected compounds such as BHT or ethanol. Lastly, significant differences in either total or specific ω-3 group proportions have been found between sample batches, as well as in certain minor components with antioxidant potential, which could entail differences in the oxidative stability of the supplements.

Regarding the oxidative status of the studied supplements, it is characterised by the predominance of primary oxidation products, with significant differences observed among samples, which are not directly correlated to their ω-3 group concentrations. This suggests that, in addition to the sample unsaturation degree, there are other factors that can have a noticeable influence on the supplement oxidative status, such as the minor component profile, but also the operations carried out and the care taken during supplement manufacture, together with the storage time and conditions.

## Figures and Tables

**Table 1:** Some composition data related to the liquid matrix of the commercial ω-3 lipid-rich supplements subject to study obtained from their labels.

Sample	Oil Nature	Some Composition Data According to the Label
S1 (c)	Cod liver oil	(*), vitamin A (from cod liver oil and retinyl palmitate)
S2 (ne)	Fish oil	In 1.14 g oil (1.25 mL): 184 mg EPA, 115 mg DHA 50% mixed tocopherols, natural lemon aroma
S3 (ne)	Tuna oil	In 4489 mg oil (5 mL): 1212 mg ω-3 lipids, 852.91 mg DHA, 197.51 mg EPA natural lemon aroma, α-tocopherol
S4 (c)	Cold-pressed linseed (*Linum usitatissimum*) oil	(*), α-tocopherol
S5 (c)	Refined ahiflower (*Buglossoides arvensis*) oil	In 1000 mg oil (1 cap): 395 mg ALA, 159 mg SDA, 83 mg LA, 41 mg GLA
S6 (c)	Echium (*Echium plantegineum*) seed oil	In 1 g oil (2 caps): 469 mg ω-3 lipids, 325 mg ALA, 144 mg SDA, 146 mg LA, 112 mg GLA
S7 (ne)	*Schizochytrium* sp. microalgae oil	50% DHA ascorbyl palmitate, natural blend of tocopherols, natural lemon aroma
S8 (c)	*Schizochytrium* sp. microalgae oil	In 1000 mg oil (2 caps): 550 mg ω-3 lipids, 300 mg DHA, 150 mg EPA D-α-tocopherol (vitamin E)
S9 (c)	Fish oil, cold-pressed borage (*Borago officinalis* L.) oil	In 400 mg fish oil (1 cap): 72 mg EPA, 48 mg DHA In 100 mg borage oil (1 cap): 22.5 mg GLA D-α-tocopherol
S10 (c)	Cold-pressed linseed oil, *Schizochytrium* sp. microalgae oil	In 600 mg lipids (1 cap): 300 mg ω-3 lipids, ≥200 mg ALA, ≥75 mg DHA natural mint essential oil, natural vitamin E
S11 (c)	Fish oil	In 2000 mg oil (2 caps): 700 mg EPA, 500 mg DHA vitamin E (D-α-tocoferol)
S12 (ne)	Concentrated fish oil (standardised 75% omega-3)	3 g ω-3 lipids (5 mL): 1.6 g EPA, 0.8 g DHA, 0.6 g other ω-3 aroma, tocopherol-rich extract
S13 (c)	Purified fish oil (50% DHA, 20% EPA)	In 500 mg oil (1 cap): Minimum 225 mg DHA (in its natural TG form), Minimum 90 mg EPA tocopherol mixture
S14 (c)	Concentrated fish oil	In 1 g fish oil (2 caps): 0.6 g ω-3 lipids (guaranteed), 0.5 g DHA, 0.1 g EPA α-tocopherol acetate (vitamin E)
S15 (c)	Purified fish oil	In 547.75 mg oil (1 cap): 465 mg ω-3 lipids, 450 mg EPA (in TG form) vitamin E (natural tocopherol blend)
S16 (c)	Purified deodorised fish oil	In 527 mg oil (1 cap): Minimum 421.6 mg ω-3 lipids, Minimum 400.5 mg DHA, Maximum 26.4 mg EPA (in TG form) blend of natural tocopherols
S17 (ne)	Fish oil (80% DHA)	In 1.02 g oil (1 mL): 800 mg DHA (in TG form), 40 mg EPA δ-tocopherol, lemon, and mint aromas

c: soft capsule; ne: non-encapsulated; cap: capsule; (*): no information on fatty acid composition declared on the label; ALA, α-linolenic acid; DHA, docosahexaenoic acid; EPA, eicosapentaenoic acid; GLA, γ-linolenic acid; SDA, stearidonic acid; LA, linoleic acid.

**Table 2:** Chemical shifts, multiplicities, and assignments of the ^1^H NMR spectral signals in CDCl_3_ of different types of protons of acyl groups/fatty acids susceptible to being present in the ω-3 lipid-rich supplement samples studied, and of the structures supporting them.

Signal	Chemical Shift (ppm)	Multiplicity	Type of Protons ^a^	Compound or Type of Compounds
A1	0.88 ^b,c^	t	-C**H_3_**	Saturated, monounsaturated ω-9 and/or ω-7 acyl groups and fatty acids
	0.89 ^b,c^	t	-C**H_3_**	Polyunsaturated ω-6 acyl groups and fatty acids
A2	0.97 ^b,c^	t	-C**H_3_**	Polyunsaturated ω-3 acyl groups and fatty acids
B	1.25 ^d^	t	ROCO-CH_2_-C**H**_3_	EE
C	1.19–1.44 ^b,c,e,f^	m *	-(C**H_2_**)_n_**-**	Acyl groups and fatty acids
D1	1.61 ^b,g^	m	-OCO-CH_2_-C**H_2_**-	Acyl groups in TG, except for DHA, DPA ω-6, EPA, ARA, SDA, and GLA groups
	1.62 ^g^	m	-OCO-CH_2_-C**H_2_**-	Acyl groups in 1,2-DG, except for DHA, DPA ω-6, EPA and ARA groups
	1.63 ^g^	m	-OCO-CH_2_-C**H_2_**-, COOH-CH_2_-C**H_2_**-	Acyl groups in 1-MG, 1,3-DG and fatty acids, except for DHA, DPA ω-6, EPA and ARA groups
	1.64 ^g^	m	-OCO-CH_2_-C**H_2_**-	Acyl groups in 2-MG, except for DHA, DPA ω-6, EPA and ARA groups
	1.65	m	-OCO-CH_2_-C**H_2_**-	SDA ** and GLA ** acyl groups in methyl ester form
D2	1.70 ^c,g^	m	-OCO-CH_2_-C**H_2_**-	EPA and ARA acyl groups in TG and EE **
	1.71	m	-OCO-CH_2_-C**H_2_**-	ARA acyl groups in methyl ester form **
	1.72 ^c,g^	m	COOH-CH_2_-C**H_2_**-	EPA and ARA (acids)
E	1.92–2.17 ^c,e,g^	m ***	-C**H_2_**-CH=CH-	Acyl groups and fatty acids except for -CH_2_- of DHA and DPA ω-6 groups in β-position in relation to the carbonyl/carboxyl group
F1	2.25–2.36 ^c,g^	dt (TG)/t (EE)	-OCO-C**H_2_**-	Acyl groups in TG and EE **, except for DHA and DPA ω-6 groups
	2.33 ^g^	m	-OCO-C**H_2_**-	Acyl groups in 1,2-DG, except for DHA and DPA ω-6 groups
	2.35 ^g^	t	-OCO-C**H_2_**-, COOH-C**H_2_**-	Acyl groups in 1-MG, 1,3-DG, and fatty acids, except for DHA and DPA ω-6 groups and EPA (acid)
	2.37/2.35 **§**	t	COOH-C**H_2_**-	EPA (acid) **
	2.38 ^g^	t	-OCO-C**H_2_**-	Acyl groups in 2-MG, except for DHA and DPA ω-6 groups
F2	2.34–2.42	m	-OCO-C**H_2_**-C**H_2_**-	DHA acyl groups in EE form **
	2.36–2.42 ^c,g^	m	-OCO-C**H_2_**-C**H_2_**-	DHA acyl groups in TG and DPA ω-6 acyl groups in methyl ester form **
	2.38–2.44 ^g^	m	COOH-C**H_2_**-C**H_2_**-	DHA and DPA ω-6 ** (acids)
G1	2.77 ^b^	t	=HC-C**H_2_**-CH=	Diunsaturated ω-6 acyl groups and fatty acids
G2	2.77–2.90 ^b,c^	m	=HC-C**H_2_**-CH=	Other polyunsaturated ω-6 and ω-3 acyl groups and fatty acids
G3	2.79–2.89	m	=HC-C**H_2_**-CH=	SDA acyl groups in methyl ester form **
H1	3.65 ^g,h^	ddd	ROCH_2_-CHOH-C**H_2_**OH	Glyceryl group in 1-MG
I1	3.73 ^g,h^	d/t **§§**	ROCH_2_-CH(OR′)-C**H_2_**OH	Glyceryl group in 1,2-DG
J1	3.84 ^g,h^	d/t **§§**	HOC**H_2_**-CH(OR)-C**H_2_**OH	Glyceryl group in 2-MG
H2	3.94 ^g,h^	m	ROCH_2_-C**H**OH-CH_2_OH	Glyceryl group in 1-MG
K	4.05–4.21 ^g,h^	m	ROC**H_2_**-C**H**OH-C**H_2_**OR’	Glyceryl group in 1,3-DG
L	4.12 ^d^	q	ROCO-C**H_2_**-CH_3_	EE
H3	4.18 ^g,h^	ddd	ROC**H_2_**-CHOH-CH_2_OH	Glyceryl group in 1-MG
M	4.22 ^b^	ddd	ROC**H_2_**-CH(OR′)-C**H_2_**OR″	Glyceryl group in TG
I2	4.28 ^g,h^	ddd	ROC**H_2_**-CH(OR′)-CH_2_OH	Glyceryl group in 1,2-DG
J2	4.93 ^g,h^	m	HOCH_2_-C**H**(OR)-CH_2_OH	Glyceryl group in 2-MG
I3	5.08 ^g,h^	m	ROCH_2_-C**H**(OR′)-CH_2_OH	Glyceryl group in 1,2-DG
N1	4.95–5.07 ^c^	dq,dq	-CH=C**H**_2_	Unsaturated ω-1 acyl groups
O	5.20–5.26 ^b^	m	ROCH_2_-C**H**(OR’)-CH_2_OR″	Glyceryl group in TG
P	5.28–5.46 ^g^	m	-C**H**=C**H**-	Acyl groups and fatty acids
N2	5.75–5.86 ^c^	m	-C**H**=CH_2_	Unsaturated ω-1 acyl groups

Abbreviations: d, doublet; ddd, doublet of double doublets; m, multiplet; t, triplet; q, quadruplet; EE, ethyl esters; TG: triglycerides; ARA, arachidonic; DHA, docosahexaenoic; DPA, docosapentaenoic; EPA, eicosapentaenoic; GLA, γ-linolenic; SDA, stearidonic; DG, diglycerides; MG, monoglycerides. * Overlapping of multiplets of methylenic protons in the different acyl groups/fatty acids either in β-position, or further, in relation to double bonds, or in γ-position, or further, in relation to the carbonyl/carboxyl group; ** Assignment on the basis of standard compounds; *** Overlapping of multiplets of the α-methylenic protons in relation to a single double bond of the different unsaturated acyl groups, except for -CH_2_- of DHA and DPA ω-6 acyl groups in β-position in relation to the carbonyl/carboxyl group; **§** This signal shows different chemical shifts if the spectrum is acquired from the pure compound or taking part in a lipid sample; **§§** This signal shows different multiplicity if the spectrum is acquired from the pure compound (t) or taking part in a lipid sample (d). **^a^** Underlined protons are those used for quantification; **^b^** Assignment taken from ref. [68]; **^c^** Assignment taken from ref. [71]; **^d^** Assignment taken from ref. [42]; **^e^** Assignment taken from ref. [69]; **^f^** Assignment taken from ref. [74]; **^g^** Assignment taken from ref. [75]; **^h^** Assignment taken from ref. [76].

**Table 3:** Molar percentages of different types of acyl groups present in the supplements studied, expressed as mean ± standard deviation (*n* = 2).

	DHA + DPA ω-6	EPA + ARA	Linolenic	SDA	Total ω-3	Other ω-3 *	Linoleic **	ω-1	Total Unsat
Fish oils									
S1	9.30 ± 0.10 ^b^	10.50 ± 0.22 ^d^	-	-	25.03 ± 0.43 ^a^	5.23 ± 0.32 ^d^	3.65 ± 0.26 ^ab^	0.95 ± 0.06 ^d^	78.23 ± 0.46 ^c^
S2	9.63 ± 0.08 ^b^	18.13 ± 0.58 ^g^	-	-	32.95 ± 0.72 ^b^	5.01 ± 0.23 ^d^	4.36 ± 0.02 ^b^	2.37 ± 0.02 ^g^	69.51 ± 0.19 ^a^
S3	24.60 ± 0.07 ^f^	7.49 ± 0.15 ^c^	-	-	33.84 ± 0.13 ^b^	1.75 ± 0.08 ^bc^	3.27 ± 0.03 ^ab^	0.08 ± 0.00 ^a^	69.46 ± 0.27 ^a^
Vegetable oils									
S4	-	-	53.49 ± 0.33 ^d^	-	***	-	14.36 ± 0.09 ^e^	-	88.26 ± 0.18 ^de^
S5	-	-	41.09 ± 0.86 ^c^	19.50 ± 0.93 ^b^	60.59 ± 0.07 ^f^	nd	12.00 ± 0.17 ^d^	-	91.86 ± 0.45 ^efg^
S6	-	-	30.03 ± 1.02 ^a^	13.93 ± 0.48 ^a^	43.96 ± 0.55 ^c^	nd	14.43 ± 0.13 ^e^	-	86.17 ± 0.39 ^d^
Microalgae oils									
S7	65.98 ± 0.01 ^j^	0.59 ± 0.01 ^a^	-	-	56.99 ± 0.25 ^e^	nd	-	-	76.22 ± 0.00 ^bc^
S8	33.37 ± 0.03 ^g^	21.40 ± 0.03 ^h^	-	-	57.64 ± 0.08 ^e^	2.87 ± 0.07 ^c^	2.91 ± 0.02 ^a^	-	74.24 ± 0.60 ^b^
Mixtures of vegetable and marine oils									
S9A	7.83 ± 0.25 ^a^	13.41 ± 0.23 ^de^	-	-	26.32 ± 0.28 ^a^	5.08 ± 0.76 ^d^	11.11 ± 1.31 ^d^	1.71 ± 0.13 ^e^	74.91 ± 1.36 ^b^
S9B	8.18 ± 0.04 ^a^	14.59 ± 0.06 ^f^	-	-	25.58 ± 0.00 ^a^	2.81 ± 0.10 ^c^	11.09 ± 0.04 ^d^	1.82 ± 0.00 ^f^	73.30 ± 0.47 ^b^
S10	15.42 ± 0.11 ^c^	1.06 ± 0.03 ^a^	34.43 ± 0.26 ^b^	-	50.91 ± 0.18 ^d^	nd	9.61 ± 0.46 ^c^	-	85.72 ± 0.34 ^d^
Fish oil concentrates									
Acyl groups in EE form									
S11	22.34 ± 0.63 ^e^	35.83 ± 0.79 ^i^	-	-	66.72 ± 0.52 ^g^	8.55 ± 0.68 ^e^	3.84 ± 0.27 ^ab^	0.35 ± 0.05 ^c^	90.20 ± 0.01 ^ef^
S12A	19.03 ± 0.69 ^d^	50.67 ± 0.83 ^l^	-	-	75.27 ± 0.26 ^i^	5.58 ± 0.12 ^d^	2.75 ± 0.24 ^a^	0.26 ± 0.00 ^bc^	95.07 ± 4.06 ^g^
S12B	19.10 ± 0.04 ^d^	46.60 ± 0.09 ^k^	-	-	71.75 ± 0.37 ^h^	6.05 ± 0.32 ^d^	2.77 ± 0.08 ^a^	0.32 ± 0.00 ^c^	91.03 ± 0.13 ^ef^
Acyl groups mainly in TG form									
S13	40.87 ± 0.02 ^h,^****	24.17 ± 0.04 ^i^	-	-	68.07 ± 0.05 ^g^	3.03 ± 0.02 ^c^	3.09 ± 0.12 ^a^	0.28 ± 0.00 ^bc^	88.83 ± 0.00 ^de^
S14	44.69 ± 0.03 ^i,^****	13.78 ± 0.35 ^ef^	-	-	72.95 ± 1.40 ^h^	14.49 ± 1.02 ^f^	-	0.30 ± 0.01 ^c^	91.06 ± 0.50 ^ef^
S15	-	88.02 ± 0.14 ^m^	-	-	88.52 ± 0.61 ^j^	0.49 ± 0.47 ^ab^	-	0.17 ± 0.00 ^ab^	92.59 ± 0.09 ^fg^
S16	80.46 ± 0.02 ^l^	6.51 ± 0.01 ^b^	-	-	92.67 ± 0.15 ^l^	5.71 ± 0.19 ^d^	-	0.07 ± 0.00 ^a^	98.15 ± 0.06 ^h^
S17A	82.42 ± 0.19 ^m^	12.51 ± 0.09 ^d^	-	-	96.65 ± 0.85 ^m^	1.71 ± 0.75 ^bc^	-	-	99.35 ± 0.47 ^h^
S17B	78.87 ± 0.17 ^k^	13.36 ± 0.25 ^de^	-	-	91.81 ± 0.81 ^k^	-	-	-	96.97 ± 0.29 ^h^

Different superscript letters within each column for each type of acyl group indicate a significant difference (*p* < 0.05) among samples. Abbreviations: DHA, docosahexaenoic; DPA, docosapentaenoic; EPA, eicosapentaenoic; ARA, arachidonic; SDA, stearidonic; Unsat, unsaturated acyl groups. * Molar percentage calculated by subtracting the sum of the molar percentages of DHA + DPA ω-6 plus EPA + ARA groups from the Total ω-3 group %; ** In the samples containing marine oils, minor diunsaturated ω-6 groups different from linoleic could be contributing to this percentage; *** Not determined because linolenic is the only type of ω-3 groups in linseed oil; **** Integration of the F2 signal of DHA groups does not include the area between 2.34 and 2.36 ppm of the potentially present DHA-EE (Table 2 and Figure 1A); -: Not detected (considered as 0 for the statistical analysis); nd: Not determinable, either because linolenic group molar percentage has been estimated by difference from the Total ω-3 group % (S5, S6 and S10 samples), or because the exact proportion of DHA groups cannot be determined due to the presence of DPA ω-6 groups (S7 sample).

**Table 4:** Data about percentages in weight of individual and total ω-3 acyl groups present in the supplements studied, taken or calculated from the information given on their respective labels, together with the differences between these data and those obtained via ^1^H NMR (Table 3), expressed as units of percentage.

	DHA	EPA	Linolenic	SDA	Total ω-3 Groups
Fish oils					
S2	10.09 (0.5↓) ^a^	16.14 (2.0↑)	-	-	-
S3	19.00 (5.6↑)	4.40 (3.1↑)	-	-	27.00 (6.8↑)
Vegetable oils					
S5	-	-	39.50 (1.6↑)	15.90 (3.6↑)	-
S6	-	-	32.50 (2.5↓)	14.40 (0.5↓)	46.90 (2.9↓)
Microalgae oils					
S7	50.00 ^b^	-	-	-	-
S8	30.00 (3.4↑)	15.00 (6.4↑)	-	-	55.00 (2.6↑)
Mixtures of vegetable and marine oils					
S9	9.60	14.40	-	-	-
	A: (1.8↓)/B: (1.4↓)	A: (1.0↓)/B: (0.2↑)			
S10	Minimum: 12.50	-	Minimum: 33.33	-	50.00 (0.9↑)
	(2.9↑)		(1.1↑)		
Fish oil concentrates					
S11	25.00 (2.7↓)	35.00 (0.8↑)	-	-	-
S12	26.67 ^c^	53.33 ^c^	-	-	75.00
	A: 25.28 ^d^ (1.4↓)	A: 67.32 ^d^ (14.0↑)			A: (0.3↑)
	B: 26.62 ^d^ (<0.1↓)	B: 64.95 ^d^ (11.6↑)			B: (3.3↓)
S13	Minimum: 45.00	Minimum: 18.00	-	-	-
	(4.1↓)	(6.2↑)			
S14	Guaranteed: 50.00	Guaranteed: 10.00	-	-	Guaranteed: 60.00
	(5.3↓)	(3.8↑)			(13.0↑)
S15	-	82.15 (5.9↑)	-	-	84.89 (3.6↑)
S16	Minimum: 76.00	Maximum: 5.00	-	-	Minimum: 80.00
	(4.5↑)	(1.5↑)			(12.7↑)
S17	78.43	3.92	-	-	-
	A: (4.0↑)/B: (0.4↑)	A: (8.6↑)/B: (9.4↑)			

Abbreviations: DHA, docosahexaenoic; EPA, eicosapentaenoic; SDA, stearidonic. -: Not present in the sample or no information available; ^a^ The value in brackets corresponds to the difference between the data from the supplement label (Table 1) and that obtained by ^1^H NMR (Table 3), expressed as units of percentage: ↑, upward difference, and ↓, downward difference; ^b^ Comparison cannot be made because the exact proportion of DHA cannot be given via ^1^H NMR due to the presence of DPA ω-6 groups; ^c^ Percentage in relation to the total of ω-3 groups; ^d^ Percentage in relation to the total of ω-3 groups calculated from data in Table 3.

**Table 5:** Concentrations of tocopherols and of certain sterols in the supplements studied, in millimoles per mole of acyl groups plus fatty acids (mmol/mol AG + FA), expressed as mean ± standard deviation (*n* = 2).

	Tocopherols			Sterols			
	α-T	γ-T	δ-T	Signal at 0.68 ppm *	DiMe-St	Stigm	∆7-Aven
Fish oils							
S1	-	-	-	3.97 ± 0.14 ^g^	-	-	-
S2	-	nq	nq	5.73 ± 0.14 ^j^	-	-	-
S3	1.81 ± 0.03 ^b,^**	1.89 ± 0.03 ^g^	0.79 ± 0.09 ^c^	1.95 ± 0.01 ^e^	-	-	-
Vegetable oils							
S4	7.95 ± 0.30 ^f^	0.39 ± 0.00 ^a^	-	1.83 ± 0.01 ^de^	1.26 ± 0.01 ^a^	nq	0.12 ± 0.00 ^a^
S5	-	0.38 ± 0.03 ^a^	-	1.58 ± 0.00 ^c^	-	-	0.19 ± 0.02 ^b^
S6	-	0.72 ± 0.01 ^b^	-	2.89 ± 0.04 ^f^	-	-	0.50 ± 0.00 ^c^
Microalgae oils							
S7	-	2.73 ± 0.02 ^h^	0.58 ± 0.00 ^b^	2.90 ± 0.00 ^f^	-	-	-
S8	4.16 ± 0.04 ^e^	0.90 ± 0.01 ^c^	0.21 ± 0.04 ^a^	0.81 ± 0.00 ^a^	-	2.87 ± 0.00	-
Mixtures of vegetable and marine oils							
S9A	3.62 ± 0.16 ^d^	-	-	5.20 ± 0.04 ^i^	-	-	-
S9B	-	3.09 ± 0.04 ^f^	1.67 ± 0.02 ^d^	4.74 ± 0.03 ^h^	-	-	-
S10	-	0.67 ± 0.08 ^b^	-	1.72 ± 0.01 ^cd^	1.41 ± 0.11 ^b^	nq	-
Fish oil concentrates							
Acyl groups in EE form							
S11	3.02 ± 0.17 ^c^	-	-	1.36 ± 0.08 ^b^	-	-	-
S12A	-	1.22 ± 0.13 ^e^	0.62 ± 0.16 ^b^	-	-	-	-
S12B	-	1.89 ± 0.02 ^g^	nq	-	-	-	-
Acyl groups mainly in TG form							
S13	-	0.90 ± 0.01 ^c^	0.51 ± 0.01 ^b^	6.20 ± 0.06 ^k^	-	-	-
S14	4.37 ± 0.05 ^e^	1.06 ± 0.02 ^d^	0.47 ± 0.02 ^b^	1.88 ± 0.04 ^de^	-	-	-
S15	0.76 ± 0.01 ^a^	0.45 ± 0.03 ^a^	nq	-	-	-	-
S16	1.68 ± 0.01 ^b^	3.87 ± 0.03 ^i^	1.83 ± 0.06 ^e^	-	-	-	-
S17A	-	-	-	-	-	-	-
S17B	-	-	-	-	-	-	-

Different superscript letters within each column for each type of compound indicate a significant difference (*p* < 0.05) among samples. Abbreviations: T, tocopherol; DiMe-St, cycloartenol and 24-methylenecycloartanol in free and/or esterified form; Stigm, stigmasterol; Aven, avenasterol. * Given by 4-desmethylsterols like cholesterol, β-sitosterol, ∆5-campesterol, and ∆5-avenasterol in free and/or esterified form; ** Mixture of α-T + α-T acetate; -: Not detected (considered as 0 for the statistical analysis); nq: Not quantified due to the presence of interfering signals in the ^1^H NMR spectrum.

**Table 6:** Concentrations of various types of minor components present in some of the supplements, in millimoles per mole of acyl groups plus fatty acids (mmol/mol AG + FA), expressed as mean ± standard deviation (*n* = 2).

	Flavour Components *					Others
	Limonene	Geranial	Neral	β-Pinene	Menthol	
S1	-	-	-	-	-	Retinyl esters: 1.01 ± 0.00
S2	22.16 ± 0.92 ^c^	1.21 ± 0.04 ^c^	0.75 ± 0.12 ^b^	2.77 ± 0.02 ^d^	-	-
S3	0.50 ± 0.01 ^a^	0.38 ± 0.00 ^a^	0.24 ± 0.01 ^a^	-	-	Ethanol: 56.12 ± 0.61
S7	0.56 ± 0.01 ^a^	-	-	-	-	BHT: 0.18 ± 0.00
S10	-	-	-	-	3.46 ± 0.24 ^a^	-
S12A	3.32 ± 0.27 ^b^	0.33 ± 0.04 ^a^	0.31 ± 0.08 ^a^	1.06 ± 0.22 ^b^	-	-
S12B	3.91 ± 0.03 ^b^	0.34 ± 0.01 ^a^	0.22 ± 0.01 ^a^	0.71 ± 0.02 ^a^	-	-
S17A	35.81 ± 0.56 ^d^	0.87 ± 0.03 ^b^	0.70 ± 0.01 ^b^	1.66 ± 0.05 ^c^	14.07 ± 0.26 ^b^	-
S17B	94.15 ± 1.25 ^e^	3.03 ± 0.00 ^d^	1.85± 0.03 ^c^	4.55 ± 0.11 ^e^	37.62 ± 0.22 ^c^	-

* These compounds were not detected in the S4, S5, S6, S8, S9A, S9B, S11, S13, S14, S15, and S16 samples. Different superscript letters within each column for each type of compound indicate a significant difference (*p* < 0.05) among samples. Abbreviations: BHT, butyl-hydroxytoluene; -: Not detected (considered as 0 for the statistical analysis).

**Table 7:** Concentrations of hydroperoxides with conjugated (*Z,E*)-dienes (HPO-c-*Z,E*-dEs), hydroxides with conjugated (*Z,E*)-dienes (HO-c-*Z,E*-dEs), and aldehydes in some of the studied supplements, in millimoles per mole of acyl groups plus fatty acids (mmol/mol AG + FA), expressed as mean ± standard deviation (*n* = 2).

	HPO-c-*Z,E*- dEs	HO-c-*Z,E*- dEs	Aldehydes				
			n-alk	2*E*-alkn	2*E*,4*E*- alkdn	2*Z*,4*E*- alkdn	Non- Identified *
S1	-	-	-	-	-	-	-
S2	0.57 ± 0.09 ^ab^	-	0.14 ± 0.01 ^b^	-	-	-	-
S3	0.47 ± 0.04 ^ab^	-	-	-	-	-	-
S4	0.42 ± 0.09 ^a^	1.61 ± 0.10 ^b^	0.10 ± 0.01 ^a^	-	-	-	-
S5	1.61 ± 0.03 ^e^	-	-	-	-	-	-
S6	2.57 ± 0.11 ^f^	-	-	0.07 ± 0.01	0.09 ± 0.01	0.07 ± 0.01 ^a^	-
S7	1.27 ± 0.15 ^cd^	-	-	-	-	-	0.32 ± 0.09 ^a^
S8	0.36 ± 0.06 ^a^	-	-	-	-	-	-
S9A	1.14 ± 0.02 ^b^	-	-	-	-	-	-
S9B	0.75 ± 0.06 ^cd^	-	-	-	-	-	-
S10	-	0.72 ± 0.01 ^a^	-	-	-	-	-
S11	2.58 ± 0.19 ^f^	-	-	-	-	-	-
S12A	-	-	-	-	-	-	-
S12B	-	-	-	-	-	-	-
S13	0.62 ± 0.02 ^ab^	-	-	-	-	-	-
S14	1.05 ± 0.15 ^c^	-	-	-	-	0.11 ± 0.00 ^b^	-
S15	1.34 ± 0.02 ^d^	-	-	-	-	-	-
S16	2.59 ± 0.03 ^f^	-	-	-	-	-	0.31 ± 0.08 ^a^
S17A	3.87 ± 0.07 ^g^	-	0.10 ± 0.01 ^a^	-	-	-	0.57 ± 0.02 ^c^
S17B	4.18 ± 0.08 ^h^	-	0.36 ± 0.04 ^c^	-	-	-	0.46 ± 0.01 ^b^

Different superscript letters within each column for each type of oxidation compound indicate a significant difference (*p* < 0.05) among samples. Abbreviations: alk, alkanals; alkn, alkenals; alkdn, alkadienals. * This group of non-identified aldehydes probably includes 2*Z*,4*E*-alkadienals; -: Not detected (considered as 0 for the statistical analysis).

## Data Availability

The original contributions presented in this study are included in the article/Supplementary Material. Further inquiries can be directed to the corresponding author.

## Supplementary Materials

The following supporting information can be downloaded at: https://www.mdpi.com/article/10.3390/foods14244217/s1. Table S1: Chemical shifts, multiplicities and assignments of the ^1^H NMR spectral signals in CDCl_3_ of different types of protons of minor components detected in the ω-3 lipid-rich supplement samples studied; Table S2: Chemical shifts, multiplicities and assignments of the ^1^H NMR spectral signals in CDCl_3_ of different types of protons of oxidation derivatives detected in the ω-3 lipid-rich supplement samples studied; Quantification from the ^1^H NMR spectral data of several compounds present in the samples; Standard compounds used for identification and quantification purposes; Figure S1: Full ^1^H NMR spectra of various ω-3 lipid standard compounds; Figure S2: Full ^1^H NMR spectra of various ω-3 and ω-6 lipid standard compounds; Figure S3: Full ^1^H NMR spectrum of the 1,3-dilinoleoyl-*rac*-glycerol (1,3-dilinolein) standard compound; Figure S4: Enlargement of the ^1^H NMR spectrum of the ARA methyl ester (ME) standard compound where its C signal appears, and the same region for the S15 sample spectrum; Figure S5: Enlargement of the ^1^H NMR spectrum of the DPA ω-3 methyl ester (ME) standard compound where its C signal appears, and the same region for some supplement sample spectra.
