# Supplementary material for: A Comprehensive Study via Proton Nuclear Magnetic Resonance of a Variety of Omega-3 Lipid-Rich Supplements Available in the Spanish Market: Acyl Group Profile, Minor Components, and Oxidative Status
Source: Foods. 2025 Dec 8;14(24):4217. doi: 10.3390/foods14244217 (PMC12731765; doi:10.3390/foods14244217)
Supplement: Supplementary file 1 [file foods-14-04217-s001.zip › foods-3912840-supplementary.pdf]

SUPPLEMENTARY MATERIAL

A Comprehensive Study via Proton Nuclear Magnetic Resonance of a Variety of Omega-3 Lipid-Rich Supplements Available in the Spanish Market: Acyl Group Profile, Minor Components, and Oxidative Status

Dafne Denise Weinbinder; María J. Manzanos; Patricia Sopelana*

Food Technology. Faculty of Pharmacy. Lascaray Research Center. University of the Basque Country (UPV/EHU). Paseo de la Universidad, 7, 01006 Vitoria-Gasteiz, Spain.

*E-mail: patricia.sopelana@ehu.eus

Table S1. Chemical shifts, multiplicities and assignments of the ^{1}H NMR spectral signals in $CDCl_3$ of different types of protons of minor components detected in the ω-3 lipid-rich supplement samples studied.

Chemical Shift (ppm)	Multiplicity	Type of Protons *	Compound or Type of Compounds
Tocopherols [a]			
1.69–1.87	m	$-CH_2-$ (C-3, ring)	α-Tocopherol / α-Tocopherol acetate
1.98	s	$-CH_3$ (bonded to C-5, aromatic ring)	α-Tocopherol acetate
2.02	s	$-CH_3$ (bonded to C-7, aromatic ring)	α-Tocopherol acetate
2.09	s	$-CH_3$ (bonded to C-8, aromatic ring)	α-Tocopherol acetate
2.11		$-CH_3$ (bonded to C-5 and C-8, aromatic ring)	α-Tocopherol
2.15	s	$-CH_3$ (bonded to C-7, aromatic ring)	α-Tocopherol
2.59	t	-C<u>**H**</u>$_2$- (C-4, aromatic ring)	α-Tocopherol acetate
2.60	t	-C<u>**H**</u>$_2$- (C-4, aromatic ring)	α-Tocopherol
6.36	s	-C<u>**H**</u>- (C-5, aromatic ring)	γ-Tocopherol
6.37	d	-C<u>**H**</u>- (C-5, aromatic ring)	δ-Tocopherol
Sterols			
0.33 [b]	d	-C<u>**H**</u>$_2$- (exo, C-19, cyclopropane ring)	Cycloartenol/24-Methylenecycloartanol
0.34 [b,c]	d	-C<u>**H**</u>$_2$- (C-19, cyclopropane ring)	Esters of Cycloartenol/24-Methylenecycloartanol
0.54 [d]	s	-C<u>**H**</u>$_3$ (C-18)	Δ7-Avenasterol (free and/or esterified)
0.55 [b]	d	$-CH_2-$ (endo, C-19, cyclopropane ring)	Cycloartenol/24-Methylenecycloartanol
0.57 [b,c]	d	$-CH_2-$ (C-19, cyclopropane ring)	Esters of Cycloartenol/24-Methylenecycloartanol
0.68 [e]	s	-C<u>**H**</u>$_3$ (C-18)	β-Sitosterol, Δ5-Campesterol, Δ5-Avenasterol, Cholesterol (free and/or esterified)
0.70 [f]	s	-C<u>**H**</u>$_3$ (C-18)	Δ5-Stigmasterol (free and/or esterified)
3.27 [b]	m	>CHOH (C-3)	Cycloartenol/24-Methylenecycloartanol
Compounds from flavouring agents			
1.65 [g]	s	$-CH_3$ (C-7)	Limonene
1.73 [g]	br s	$-CH_3$ (C-10)	Limonene
4.70 [g]	br s	-C<u>**H**</u>$_2$ (C-9)	Limonene
3.42 [h]	dt	-C<u>**H**</u>OH (C-1α)	Menthol
1.64 [h]	dtd	-CH (C-5α)	Menthol
1.58 [h]	qd	-CH (C-4α)	Menthol
4.55 [i]	m	-C<u>**H**</u>s (C10)	β-Pinene
4.62 [i]	m	-CHa (C10)	β-Pinene
9.89 [i]	d	-C<u>**H**</u>O	Neral
9.99 [i]	d	-C<u>**H**</u>O	Geranial

Table S1. Continuation.

Chemical Shift (ppm)	Multiplicity	Type of Protons *	Compound or Type of Compounds
Vitamin A [j]			
1.89	s	-C**H**$_3$ (C-19)	Retinol/retinyl esters
4.32	d	=CH-C**H**$_2$OH (C-15)	Retinol
4.69	d	=CH-C$\underline{\mathbf{H}_2}$OCOR (C-15)	Retinyl esters
6.29	d	-CH=C**H**- (C-12)	Retinol/retinyl esters
6.61	dd	-C**H**=CH- (C-11)	Retinol/retinyl esters
Other minor compounds			
3.71 [k]	q	CH$_3$-C$\underline{\mathbf{H}_2}$OH	Ethanol
1.25 [k]	t	**CH**$_3$-CH$_2$OH	Ethanol
6.97 [l]	**s**	-C$\underline{\mathbf{H}}$=C(CH$_3$)-C$\underline{\mathbf{H}}$= (C-3, C-5)	2,6-Di-*tert*-butyl-hydroxytoluene (BHT)

Abbreviations: br, broad; d, doublet; dd, double doublet; m, multiplet; q, quadruplet; s, singlet; t, triplet.

* Underlined protons are those used for quantification.

[a] Assignment taken from Baker, J.K.; Myers, C.W. One-Dimensional and Two-Dimensional ^{1}H-and ^{13}C-Nuclear Magnetic Resonance (NMR) Analysis of Vitamin E Raw Materials or Analytical Reference Standards. *Pharm. Res.* **1991**, *8*, 763-770 [ref. 77. in the main text].

[b] Assignment taken from Ruiz-Aracama, A.; Goicoechea, E.; Guillén, M.D. Direct study of minor extra-virgin olive oil components without any sample modification. ^{1}H NMR multisupression experiment: A powerful tool. *Food Chem.* **2017**, *228*, 301-314 [ref. 78 in the main text].

[c] Assignment taken from Bouvier-Navé, P.; Husselstein, T.; Benveniste, P. Two families of sterol methyltransferases are involved in the first and the second methylation steps of plant sterol biosynthesis. *Eur. J. Biochem.* **1998**, *256*, 88-96 [ref. 79 in the main text].

[d] Assignment taken from Zhang, X.; Cambrai, A.; Miesch, M.; Roussi, S.; Raul, F.; Aoude-Werner, D.; Marchioni, E. Separation of Δ5- and Δ7-Phytosterols by Adsorption Chromatography and Semipreparative Reversed Phase High-Performance Liquid Chromatography for Quantitative Analysis of Phytosterols in Foods. *J. Agric. Food Chem.* **2006**, *54*, 1196-1202 [ref. 80 in the main text].

[e] Assignments taken from:

Zhang et al., **2006** [ref. 80 in the main text] (β-Sitosterol, Δ5-Campesterol and Δ5-Avenasterol).

Guillén, M.D.; Carton, I.; Goicoechea, E.; Uriarte, P.S. Characterization of Cod Liver Oil by Spectroscopic Techniques. New Approaches for the Determination of Compositional Parameters, Acyl Groups, and Cholesterol from ^{1}H Nuclear Magnetic Resonance and Fourier Transform Infrared Spectral Data. *J. Agric. Food Chem.* **2008**, *56*, 9072-9079 [ref. 81 in the main text] (Cholesterol).

[f] Assignment taken from Forgo, P.; Kövér, K.E. Gradient enhanced selective experiments in the ^{1}H NMR chemical shift assignment of the skeleton and side-chain resonances of stigmasterol, a phytosterol derivative. *Steroids* **2004**, *69*, 43-50 [ref. 82 in the main text].

[g] Assignment taken from Santos, J.S.; Escher, G.B.; da Silva Pereira, J.M.; Marinho, M.T.; do Prado-Silva, L.; Sant'Ana, A.S.; Dutra, L.D.; Barison, A.; Granato, D. ^{1}H NMR combined with chemometrics tools for rapid characterization of edible oils and their biological properties. *Ind. Crops Prod.* **2018**, *116*, 191-200 [ref. 83 in the main text].

[h] Assignment taken from Atta-ur-Rahman, A.; Yaqoob, M.; Farooq, A.; Anjum, S.; Asif, F.; Choudhary, M.I. Fungal Transformation of (1*R*,2*S*,5*R*)-(−)-Menthol by *Cephalosporium aphidicola*. *J. Nat. Prod.* **1998**, *61*, 1340-1342 [ref. 84 in the main text].

[I] Assignment taken from Salvino, R.A.; Aroulanda, C.; De Filpo, G.; Celebre, G.; De Luca, G. Metabolic composition and authenticity evaluation of bergamot essential oil assessed by nuclear magnetic resonance spectroscopy. *Anal. Bioanal. Chem.* **2022**, *414*, 2297-2313 [ref. 85 in the main text].

[j] Assignments taken from Choi, Y.H.; Kim, H.K.; Wilson, E.G.; Erkelens, C.; Trijzelaar, B.; Verpoorte, R. Quantitative analysis of retinol and retinol palmitate in vitamin tablets using ^{1}H-nuclear magnetic resonance spectroscopy. *Anal. Chim. Acta* **2004**, *512*, 141-147 [ref. 86 in the main text].

[k] Assignment taken from Gottlieb, H.E.; Kotlyar, V.; Nudelman, A. NMR Chemical Shifts of Common Laboratory Solvents as Trace Impurities. *J. Org. Chem.* **1997**, *62*, 7512-7515 [ref. 87 in the main text].

[l] Assignment taken from Nieva-Echevarría, B.; Goicoechea, E.; Guillén, M.D. Polyunsaturated lipids and vitamin A oxidation during cod liver oil *in vitro* gastrointestinal digestion. Antioxidant effect of added BHT. *Food Chem.* **2017**, *232*, 733-743 [ref. 88 in the main text].

Table S2. Chemical shifts, multiplicities and assignments of the ^{1}H NMR spectral signals in $CDCl_3$ of different types of protons of oxidation derivatives detected in the ω-3 lipid-rich supplement samples studied.

Chemical Shift (ppm)	Multiplicity	Type of Protons *	Compound or Type of Compounds
6.47 [a]	dd	-CH=CH-C**H**=CH-CHOH-	*Z*,*E*-conjugated double bonds associated with an hydroxy group in octadecadienoic acyl chains (13-hydroxy-9*Z*,11*E*-octadecadienoic acid)
6.49 [b] / 6.52 [c] (*Z*,*E*)<br>6.53 [c] (*E*,*Z*)	dd	-CH=CH-C**H**=CH-CHOH-<br>(*Z*,*E*)<br>-CHOH-CH=C**H**-CH=CH-<br>(*E*,*Z*)	*Z*,*E*/*E*,*Z*-conjugated double bonds associated with an hydroxy group in octadecatrienoic acyl chains<br>9*S*-hydroxy-10*E*,12*Z*,15*Z*-octadecatrienoic acid**
6.58 [d]	dddd	-C**H**=CH-CHOOH-	*Z*,*E*-conjugated double bonds associated with an hydroperoxy group in octadecadienoic acyl chains
6.54 [b] / 6.62 [e] (*Z*,*E*)<br>6.61 [e] (*E*,*Z*)<br>(6.59) ***	dddd	-CH=CH-C**H**=CH-CHOOH-<br>(*Z*,*E*)<br>-CHOOH-CH=C**H**-CH=CH-<br>(*E*,*Z*)	*Z*,*E*/*E*,*Z*-conjugated double bonds associated with an hydroperoxy group in octadecatrienoic acyl chains<br>13-hydroperoxy-9*Z*,11*E*,15*Z*-octadecatrienoic acid**
9.49 [d,f]	d	-C**H**O	2*E*-alkenals
9.53 [d,f]	d	-C**H**O	2*E*,4*E*-alkadienals
9.59 [g]	d	-C**H**O	2*Z*,4*E*-alkadienals
9.75 [d,f]	t	-C**H**O	n-alkanals with more than 3 carbon atoms

Abbreviations: d, doublet; dd, double doublet; t, triplet.

* Underlined protons are those used for quantification; **Standard compound; *** Value in parenthesis corresponds to the chemical shift of the standard compound, which agrees with that in the sample spectra.

[a] Assignment taken from Manini, P.; Camera, E.; Picardo, M.; Napolitano, A.; d'Ischia, M. Free radical oxidation of coriolic acid (13-(*S*)-hydroxy-9*Z*,11*E*-octadecadienoic Acid). *Chem. Phys. Lipids* **2005**, *134*, 161-171 [ref. 89 in the main text].

[b] Assignment taken from Gardner, H.; Weisleder, D. Hydroperoxides from Oxidation of Linoleic and Linolenic Acids by Soybean Lipoxygenase: Proof of the *trans*-11 Double Bond. *Lipids* **1972**, *7*, 191-193 [ref. 90 in the main text] (13-hydroxy-9*Z*,11*E*,15*Z*-octadecatrienoic acid and 13-hydroperoxy-9*Z*,11*E*,15*Z*-octadecatrienoic acid).

[c] Assignment taken from Kikuchi, M.; Yaoita, Y.; Kikuchi, M. Monohydroxy-Substituted Polyunsaturated Fatty Acids from *Swertia japonica*. *Helv. Chim. Acta* **2008**, *91*, 1857-1862 [ref. 91 in the main text] (16*R*-hydroxy-9*Z*,12*Z*,14*E*-octadecatrienoate and 12*S*-hydroxy-9*Z*,13*E*,15*Z*-octadecatrienoate).

[d] Assignment taken from Goicoechea, E.; Guillén, M.D. Analysis of Hydroperoxides, Aldehydes and Epoxides by ^{1}H Nuclear Magnetic Resonance in Sunflower Oil Oxidized at 70 and 100 ºC. *J. Agric. Food Chem.* **2010**, *58*, 6234-6245 [ref. 92 in the main text].

[e] Assignment taken from Ahmed, R.; Varras, P.C.; Siskos, M.G.; Siddiqui, H.; Choudhary, M.I.; Gerothanassis, I.P. NMR and Computational Studies as Analytical and High-Resolution Structural Tool for Complex Hydroperoxides and Diastereomeric *Endo*-Hydroperoxides of Fatty Acids in Solution-Exemplified by Methyl Linolenate. *Molecules*, **2020**, *25*, 4902 [ref. 93 in the main text] (16-hydroperoxy-9*Z*,12*Z*,14*E*-octadecatrienoate and 9-hydroperoxy-10*E*,12*Z*,15*Z*-octadecatrienoate).

[f] Assignment taken from Guillén, M.D.; Ruiz, A. Formation of hydroperoxy- and hydroxyalkenals during thermal oxidative degradation of sesame oil monitored by proton NMR. *Eur. J. Lipid Sci. Technol*. **2004**, *106*, 680-687 [ref. 94 in the main text].

[g] Assignment taken from Guillén, M.D.; Ruiz, A. Monitoring the oxidation of unsaturated oils and formation of oxygenated aldehydes by proton NMR. *Eur. J. Lipid Sci. Technol*. **2005**, *107*, 36-47 [ref. 95 in the main text].

Quantification from the ^{1}H NMR spectral data of several compounds present in the samples

Bearing in mind that the area of each ^{1}H NMR spectral signal is proportional to the number of protons that generate it, and that the proportionality constant is the same for all kinds of protons, the area of some spectral signals can be employed to quantify a wide variety of compounds, as detailed below.

Molar percentages of different types of acyl groups/fatty acids

Total ω-3 group % = $100 \times [(4A_{A2}/(6A_{F1}+3A_{F2})]$ (equation S1)

DHA group % = $100 \times [A_{F2}/(2A_{F1}+A_{F2})]$ (equation S2)

EPA+ARA group % = $100 \times [2A_{D2}/(2A_{F1}+A_{F2})]$ (equation S3)

Linolenic group % = $100 \times (A_{G2}/2A_{F1})$ (equation S4)*

Linoleic (diunsaturated ω-6) group % = $100 \times [2A_{G1}/(2A_{F1}+A_{F2})]$ (equation S5)**

ω-1 group % = $100 \times [2A_{N1}/(2A_{F1}+A_{F2})]$ (equation S6)

Total unsaturated group % = $100 \times [(2A_{E}+A_{F2})/(4A_{F1}+2A_{F2})]$ (equation S7)

where A_{A2}, A_{F1}, A_{F2}, A_{D2}, A_{G2}, A_{G1}, A_{N1} and A_{E} are the areas of signals A2, F1, F2, D2, G2, G1, N1 and E, respectively (signals and assignments in Table 2).

*Equation S4 can only be used to calculate linolenic group molar percentages in samples free of both stearidonic groups and long-chain polyunsaturated groups such as eicosapentaenoic (EPA, ω-3), docosapentaenoic (DPA, ω-3), docosahexaenoic (DHA, ω-3) and arachidonic (ARA, ω-6).

**If DHA groups are not present in the sample, the equation will be simplified as follows:
Linoleic group % = $100 \times (A_{G1}/A_{F1})$

It must be noted that, in the case of EPA+ARA acyl group molar percentage determination, different factors calculated from the EPA standard compounds had to be applied to estimate the total area of signal D2 in several samples. The reason was that only a part of this signal was integrated due to its partial overlap with signal D1 and/or with other unknown signals. Likewise, due to the partial overlap of G1 and G2 signals, both areas must be corrected to calculate linoleic and linolenic molar percentages, respectively. For this purpose, trilinolein and trilinolenin were used as references.

Concentration of different types of minor components, expressed in millimoles per mole of acyl groups plus fatty acids (mmol/mol AG + FA)

[C] (mmol/mol AG + FA) = $1000 \times [(A_{S}/n)/((A_{F1}/2)+(A_{F2}/4))]$ (equation S8)

where A_{S} is the area of the signal used to quantify each type of compound (C) and n the number of protons that generate each signal.

Similarly to that explained for the calculation of certain acyl group molar percentages, some corrections had to be made to calculate the area of the signals of certain minor components in some samples. This is the case of α-tocopherol, as the signal selected for its quantification (Table S1) overlaps with the side-band of *bis*-allylic proton signal G1 (Table 2), and of δ-tocopherol, whose quantifiable signal overlaps with that of γ-tocopherol (Table S1). Consequently, correction factors to estimate the total area of the quantifiable signals were calculated from the spectra of the corresponding standard compounds.

Standard compounds used for identification and quantification purposes

4*Z*,7*Z*,10*Z*,13*Z*,16*Z*,19*Z*-Docosahexaenoic acid (DHA) and 5*Z*,8*Z*,11*Z*,14*Z*,17*Z*-eicosapentaenoic acid (EPA) ethyl ester (Biosynth, Staad, Switzerland); DHA ethyl ester and D-α-tocopherol acetate (TCI, Zwijndrecht, Belgium); 4*Z*,7*Z*,10*Z*,13*Z*,16*Z*-docosapentaenoic acid (DPA ω-6), DPA ω-6 methyl ester, 7*Z*,10*Z*,13*Z*,16*Z*,19*Z*-docosapentaenoic acid (DPA ω-3) methyl ester, 5*Z*,8*Z*,11*Z*,14*Z*-eicosatetraenoic acid (arachidonic acid, ARA) methyl ester, 6*Z*,9*Z*,12*Z*,15*Z*-octadecatetraenoic acid (stearidonic acid, SDA) methyl ester, 6*Z*,9*Z*,12*Z*-octadecatrienoic acid (gamma-linolenic acid, GLA) methyl ester, tridocosahexaenoin, trieicosapentaenoin, trilinolenin, trilinolein, Δ5-campesterol and Δ7-Avenasterol (Larodan, Solna, Sweeden) were acquired through the Spanish distributor Cymit Quimica (Barcelona, Spain).

1,2-Diolein, 1,3-dilinoleoyl-*rac*-glycerol (1,3-dilinolein), 1-monolinolein, 2-monoolein, 2-monopalmitin, ethyl arachidonate, D-α-tocopherol, δ-tocopherol, β-sitosterol, Δ5-stigmasterol, cycloartenol, cholesterol, β-pinene, menthol, retinol, retinyl acetate, retinyl palmitate, hexanal, 2*E*-hexenal, 2*E*-heptenal, 2*E*-decenal, 2*E*,4*E*-hexadienal, 2*E*,4*E*-heptadienal, 2*E*,4*E*-decadienal and 2,6-di-*tert*-butylhydroxytoluene (BHT) (Sigma-Aldrich, St. Louis, MO, USA) were purchased from Merck KGaA (Darmstadt, Germany).

Citral (a mixture of geranial and neral) and (R)-(+)-limonene were purchased from Alfa Aesar (Haverhill, MA, USA), γ-tocopherol from Eisai Food & Chemical Co. (Tokyo, Japan), Δ5-Avenasterol from Wuhan ChemFaces Biochemical Co. (Wuhan, P.R. China) and absolute ethanol from Comar Araba (Vitoria-Gasteiz, Spain).

Finally, 13-hydroperoxy-9*Z*,11*E*,15*Z*-octadecatrienoic acid and 9*S*-hydroxy-10*E*,12*Z*,15*Z*-octadecatrienoic acid were acquired from Cayman Chemical (Ann Arbor, MI, USA).

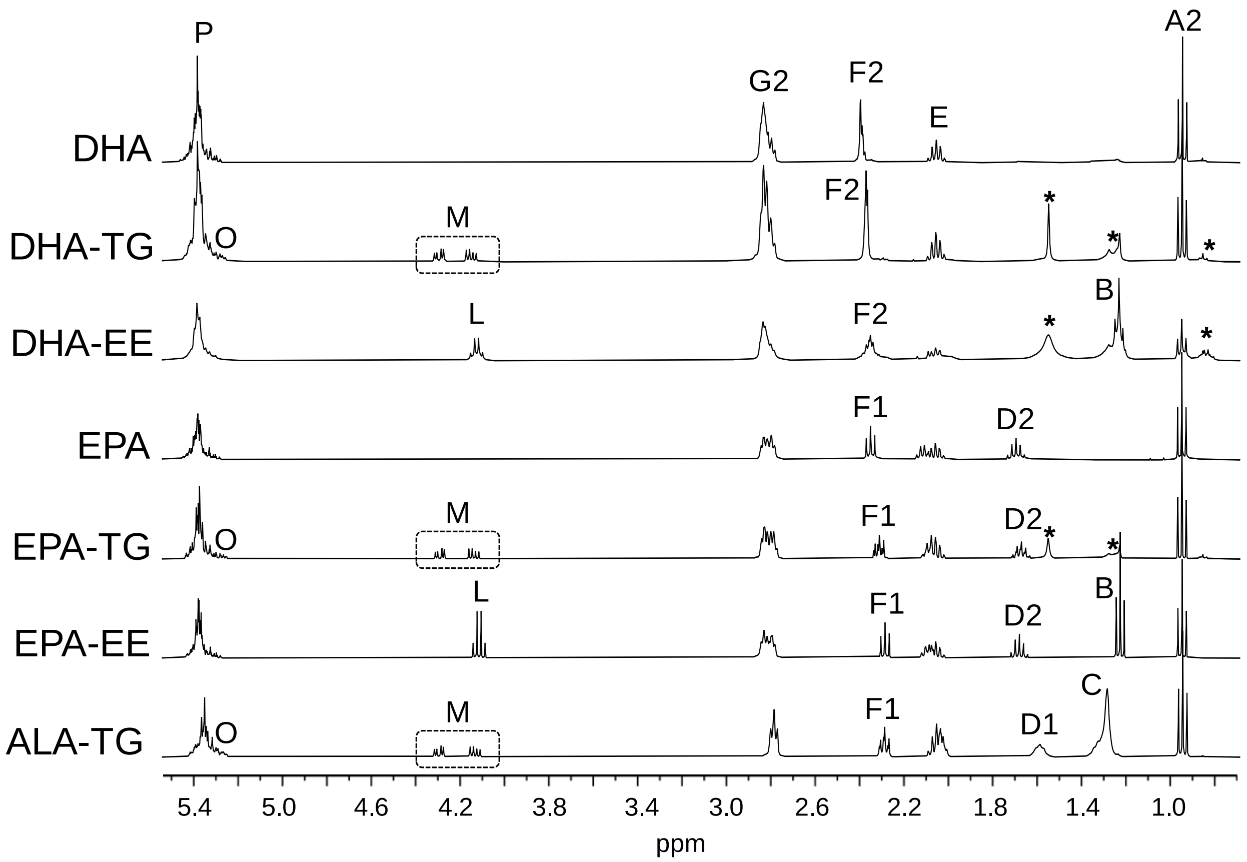

Figure S1. Full ^{1}H NMR spectra of various ω-3 lipid standard compounds. ALA: α-linolenic; DHA: docosahexaenoic; EPA; eicosapentaenoic; TG: triglyceride; EE: ethyl ester. *Signals from impurities present in the standard compound. Signal letters agree with those in Table 2.

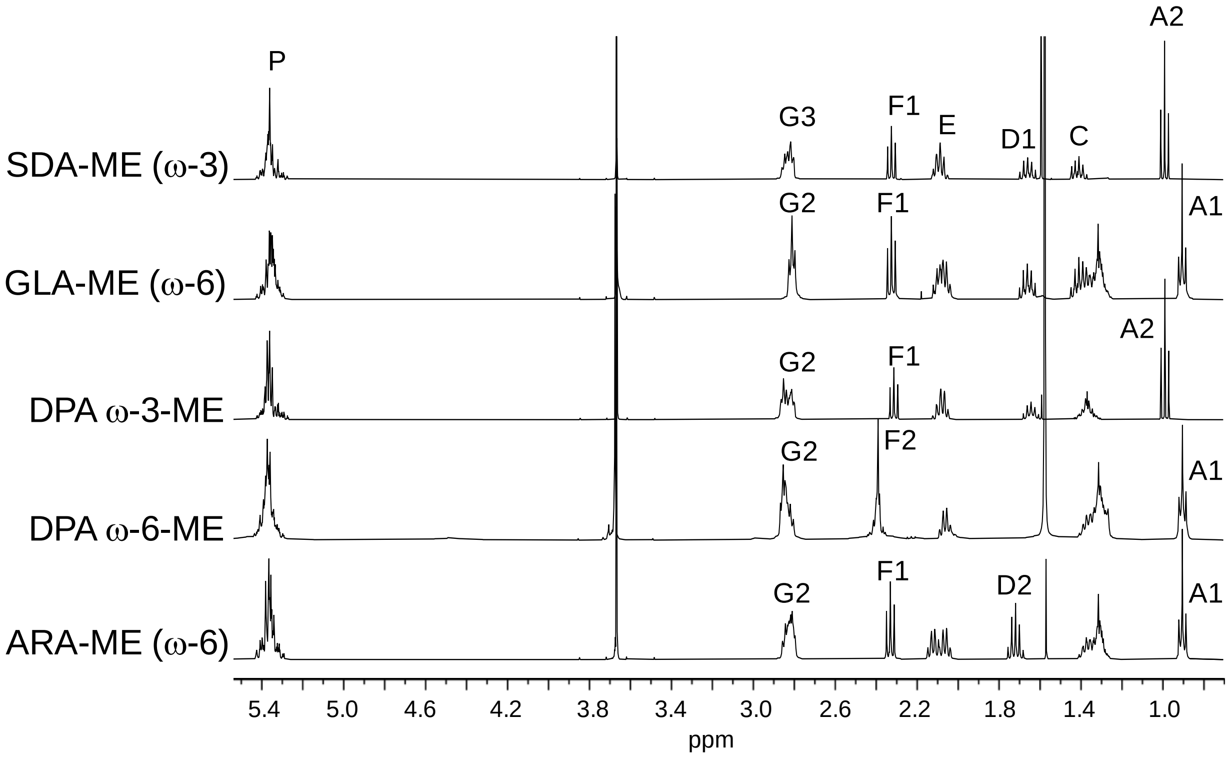

Figure S2. Full ^{1}H NMR spectra of various ω-3 and ω-6 lipid standard compounds. ARA: arachidonic; DPA: docosapentaenoic; GLA: γ-linolenic; SDA: stearidonic; ME: methyl ester. Signal letters agree with those in Table 2.

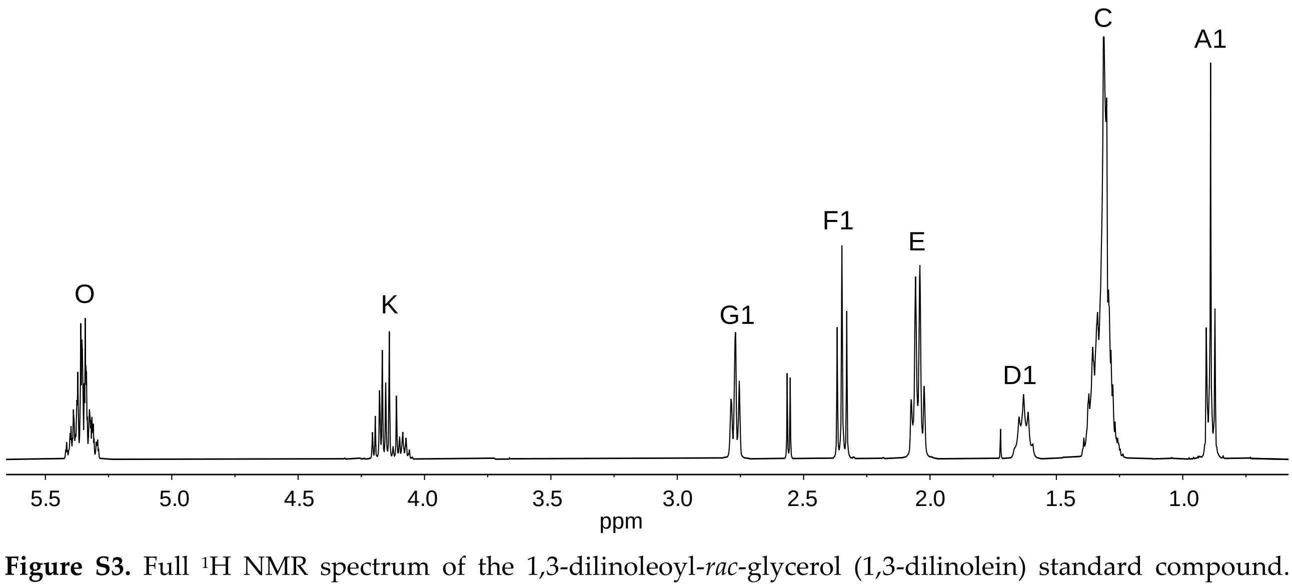

Figure S3. Full ^{1}H NMR spectrum of the 1,3-dilinoleoyl-*rac*-glycerol (1,3-dilinolein) standard compound. Signal letters agree with those in Table 2.

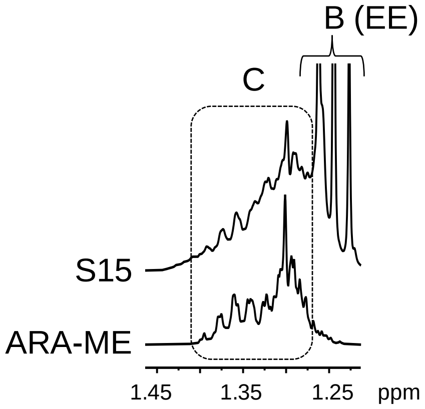

Figure S4. Enlargement of the ^{1}H NMR spectrum of the ARA methyl ester (ME) standard compound where its C signal appears (Table 2), and the same region for the S15 sample spectrum. EE: ethyl esters.

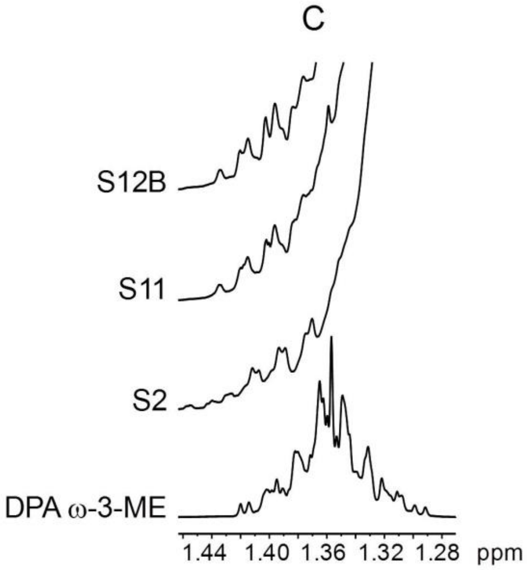

Figure S5. Enlargement of the ^{1}H NMR spectrum of the DPA ω-3 methyl ester (ME) standard compound where its C signal appears (Table 2), and the same region for some supplement sample spectra.
